# Supramolecular assembly properties of a mixed-sequence recognition-encoded melamine oligomer†

**DOI:** 10.1039/d5ob00769k

**Published:** 2025-07-04

**Authors:** Mohit Dhiman, Joseph T. Smith, Christopher A. Hunter

**Affiliations:** a Yusuf Hamied Department of Chemistry, University of Cambridge Lensfield Road Cambridge CB2 1EW UK herchelsmith.orgchem@ch.cam.ac.uk

## Abstract

Recognition-encoded melamine oligomers (REMO) are composed of an alternating piperazine-triazine backbone and side-chains equipped with either a H-bond donor (phenol, D) or a H-bond acceptor (phosphine oxide, A). Complementary homo-oligomers form stable duplexes in organic solvents, due to intermolecular base-pairing interactions between the phenol and phosphine oxide side-chains. For mixed-sequence oligomers, the major pathway that competes with duplex formation is folding due to intramolecular base-pairing interactions. Automated solid phase synthesis was used to prepare the self-complementary REMO DADA, and this oligomer was used to investigate the competition between intermolecular and intramolecular H-bonding interactions. Isothermal titration calorimetry in chloroform showed that DADA forms a dimeric complex, but with reduced stability compared with the duplexes formed by shorter oligomers. The results indicate that a folded state with intramolecular interactions between the two terminal recognition units is significantly populated. The dimeric complex formed at higher concentrations could involve the interaction of two folded oligomers in a kissing stem-loops structure, or the oligomer could unfold to give the duplex with four intermolecular base-pairs. One end of the oligomer was equipped with an azide and the other with an alkyne, so that the dimeric complex could be covalently trapped using copper-catalysed azide–alkyne cycloaddition reactions. The major product was the macrocyclic duplex with small amounts of the macrocyclic single-strand, which shows that the DADA·DADA duplex dominates at millimolar concentrations. Understanding the propensity of the REMO architecture to fold will help guide the future design principles for synthesis of more complex functional assemblies.

## Abstract 2

The self-complementary REMO with sequence DADA was prepared by automated solid phase synthesis. Folding due to intramolecular H-bonding competes with duplex formation due to intermolecular phenol·phosphine oxide base-pairing interactions.

## Graphical abstract

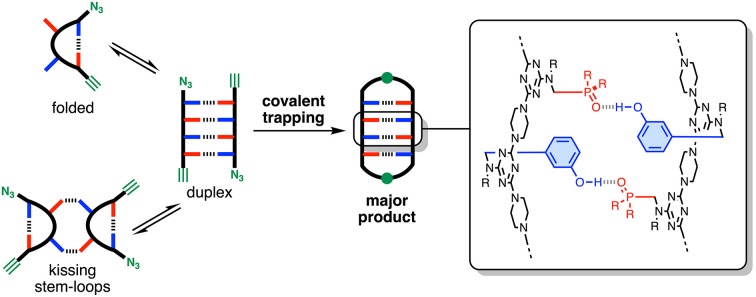

## Introduction

**Fig. 1:**
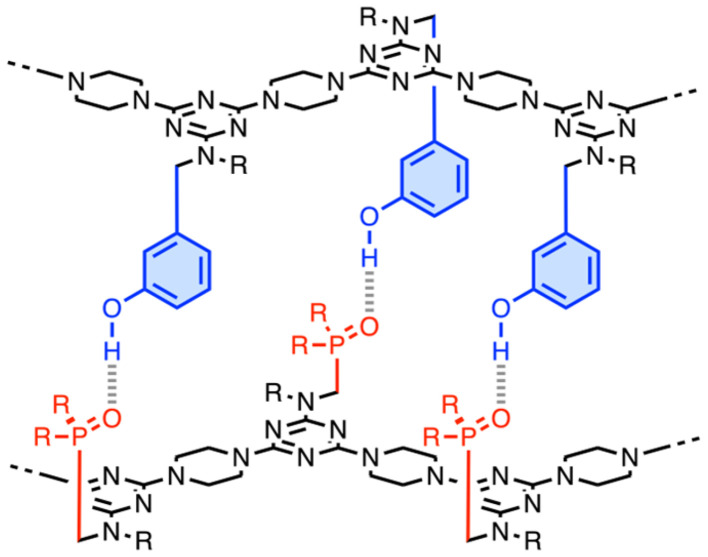
Phenol·phosphine oxide H-bonding interactions lead to the assembly of duplexes between complementary sequences of recognition-encoded melamine oligomers (REMO). R = isobutyl or 2-ethylhexyl.

The functional properties of biological polymers are encoded by the sequence of monomer building blocks. In nucleic acids, sequence-complementary strands form stable duplexes due to hydrogen-bonding interactions between nucleobases,^1,2^ and this structure is the basis for molecular replication and translation of sequence information in living systems. The sequences of proteins determine how they fold into three-dimensional structures, which in turn dictates their recognition, catalysis, and self-assembly properties.^3,4^ Synthetic polymers made from sequences of different monomer building blocks would allow exploration of new areas of chemical space for structures with functional properties.^5,6^ Inspired by DNA, chemists have developed synthetic oligomers that form duplexes based on different types of non-covalent or dynamic covalent interaction.^7–23^ We have developed Recognition-Encoded Melamine Oligomers (REMO) that feature an alternating 1,3,5-triazine-piperazine backbone and two different side-chains equipped with either a phenol (D) or phosphine oxide (A) recognition unit.^24^ The side-chains encode sequence information and carry H-bonding sites, leading to length- and sequence-selective duplex formation with remarkably high fidelity (Fig. 1).^25^

**Fig. 2:**
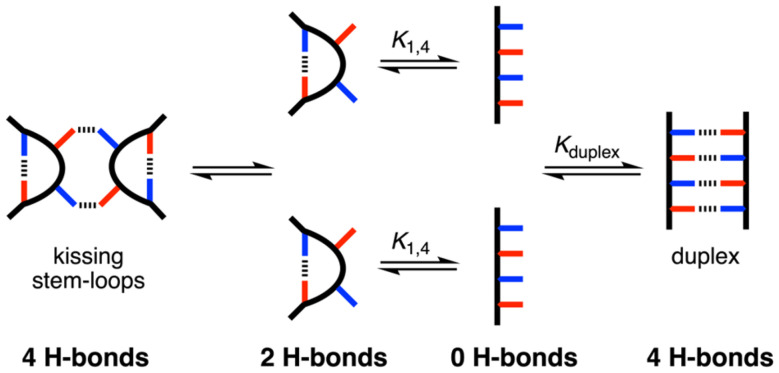
Possible folding and self-assembly pathways for a REMO with the sequence DADA.

Systems that form synthetic duplexes are generally based on the interaction between complementary homo-oligomers, but recently duplexes formed by two mixed-sequence oligomers have been reported.^24,26–30^ The self-assembly properties of these systems are more complicated, because intramolecular interactions between complementary recognition units on the same strand of a mixed-sequence oligomer can lead to folding.^31,32^ There may be multiple equilibria that compete with duplex formation, which is not the case for homo-oligomers. The REMO backbone is sufficiently rigid to prevent both 1,2-folding, due to intramolecular H-bonding between neighbouring recognition units on the same chain, and 1,3-folding.^24^ Here we describe the self-assembly properties of the REMO 4-mer sequence DADA. The sequence-complementary recognition units in the terminal positions could lead to 1,4-folding, but the sequence is also self-complementary, which should promote formation of the dimeric duplex. Fig. 2 illustrates different structures that could result. Depending on relative values of the effective molarities (EM) for the intramolecular interactions that lead to the duplex and to folding, it is also possible for the dimeric complex to adopt a kissing stem-loops structure.^33^ The competition between these equilibria was explored using isothermal titration calorimetry (ITC), NMR spectroscopy, and *in situ* covalent trapping of self-assembled structures using copper-catalysed azide–alkyne cycloaddition (CuAAC) reactions on oligomers functionalised with terminal azide and alkyne groups.

## Results and discussion

### Synthesis

**Fig. 3:**
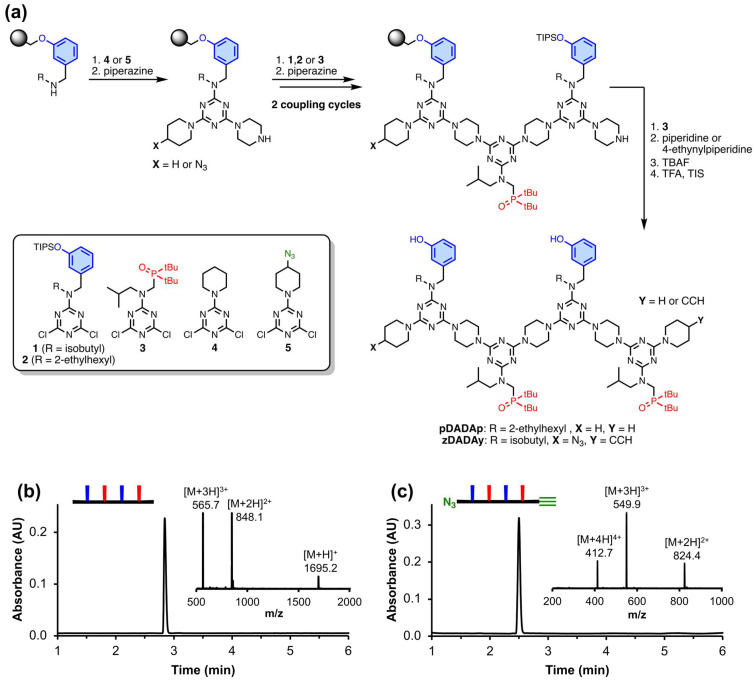
(a) Automated SPS of REMO. (b) UPLC trace and ESI-MS of the HPLC purified product, pDADAp, and cartoon representation of the structure. Calculated mass (ESI^+^): 1695.2 [M + H]^+^, 848.1 [M + 2H]^2+^, 565.7 [M + 3H]^3+^. Experimental masses are shown in the figure. (c) UPLC trace and ESI-MS of the HPLC purified product, zDADAy, and cartoon representation of the structure. Calculated mass (ESI^+^): 824.5 [M + 2H]^2+^, 550.0 [M + 3H]^3+^, 412.8 [M + 4H]^4+^. *UPLC conditions*: C4 column at 40 °C using a 30–100% gradient of THF/formic acid (0.1%) in water/formic acid (0.1%) over 4 minutes, then 100% THF/formic acid (0.1%) over 2 minutes.

**Scheme 1:**
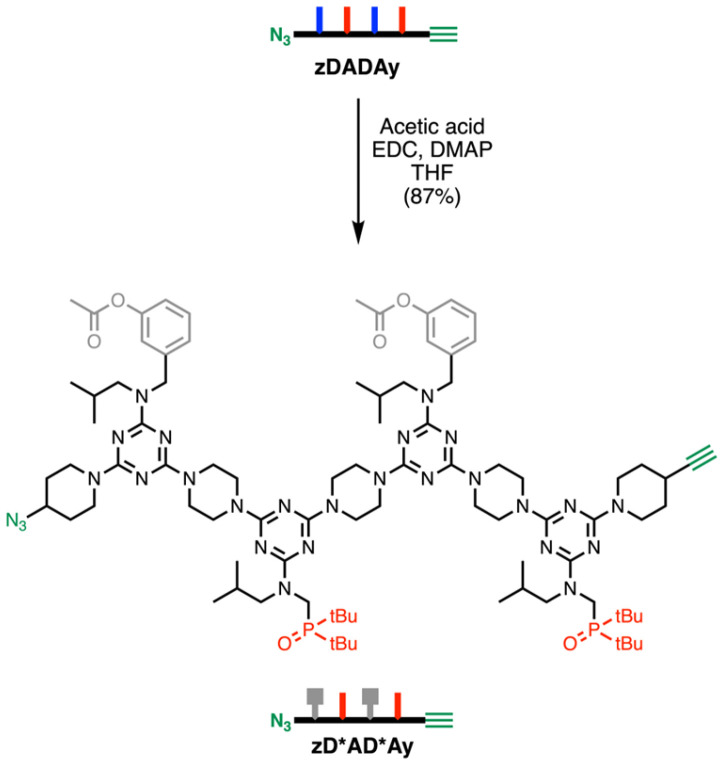
Synthesis of reference compound zD*AD*Ay. The acetylated phenol groups that do not function as recognition groups are represented in grey in the cartoon structure.

We recently described a highly efficient, automated solid-phase synthesis (SPS) method to access REMO of any desired sequence.^34^Fig. 3(a) shows the SPS route to pDADAp and zDADAy, where the oligomer sequences are described using upper-case letters for the recognition units (D for phenol, and A for phosphine oxide) and lower-case letters for the end groups (p for piperidine, z for azide, and y for alkyne). Oligomers were synthesised by iterative rounds of S_N_Ar reactions with a derivatised TentaGel Wang resin, alternately coupling with a dichlorotriazine then piperazine. In the final coupling step, the oligomer was capped with either piperidine or 4-ethynyl piperidine, followed by deprotection of the phenol groups and cleavage from the resin. HPLC was used to isolate the final products (greater than 99% purity by LCMS). Fig. 3(b) and (c) show the UPLC traces and ESI-MS of pDADAp and zDADAy respectively. The oligomers were further characterised by ^1^H and ^31^P NMR spectroscopy, and HRMS to confirm their identity (see ESI†). A sample of zDADAy was also acetylated using acetic acid to obtain zD*AD*Ay (where D* represents an acetylated phenol), which was used as a reference compound that cannot self-assemble, because the phenol recognition sites are blocked (Scheme 1).^25,33^ The monomers pAp and pDp were also prepared as reference compounds.

### Binding studies

**Fig. 4:**
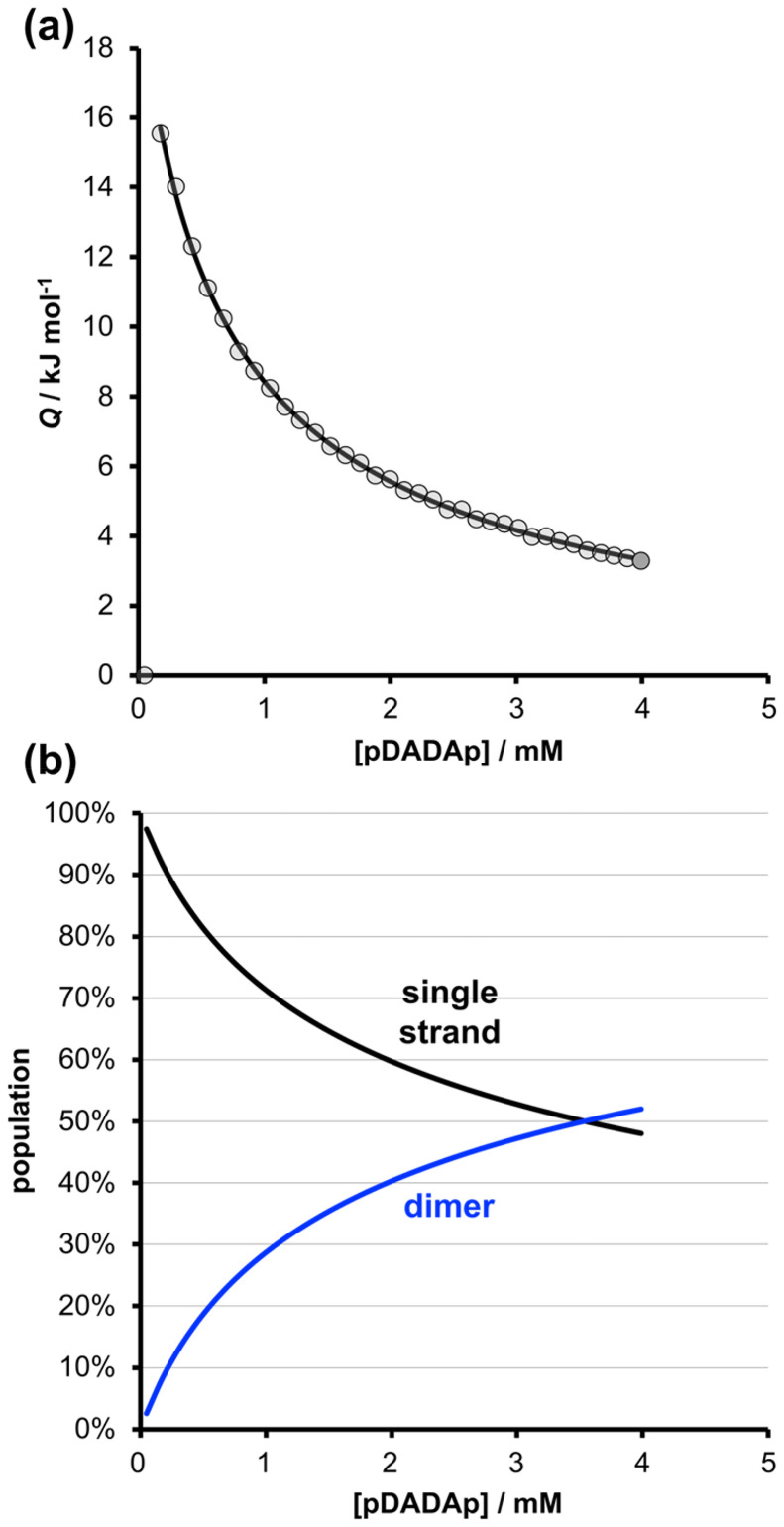
(a) Isothermal titration calorimetry data (circles) for dilution of pDADAp in chloroform solution at 298 K. The line of best fit to a dimerisation isotherm corresponds to *K* = 300 ± 50 M^−1^ and Δ*H*° = −43 ± 2 kJ mol^−1^ (errors at the 95% confidence limit). (b) Calculated speciation profile for the ITC experiment plotted as a function of pDADAp concentration in chloroform at 298 K.

ITC was used to investigate the self-association of pDADAp in chloroform. The data from dilution experiments fit well to a dimerisation isotherm with a self-association constant of *K* = 300 ± 50 M^−1^ (Fig. 4). This value can be compared with the association constant for formation of the 1 : 1 complex between the 3-mer homo-oligomers AAA and DDD, which was measured using ^31^P NMR titration experiments in chloroform (*K* = 3100 ± 100 M^−1^, see ESI† for details). We have previously shown that the stability of REMO duplexes depends on the number of intermolecular base-pairing interactions.^24^ Neither homo-oligomer AAA or DDD can fold, and the AAA·DDD duplex, which forms three intermolecular H-bonds, is an order of magnitude more stable than the pDADAp·pDADAp complex. This result indicates that less than three intermolecular H-bonds are formed when pDADAp self-associates. If the folded structure were fully populated in the monomeric single-stranded state, there would be a net gain of only two H-bonds when the dimeric pDADAp·pDADAp complex was formed. Therefore the ITC experiment shows that 1,4-folding occurs to a significant extent in the single-stranded form of pDADAp (see Fig. 2).

**Fig. 5:**
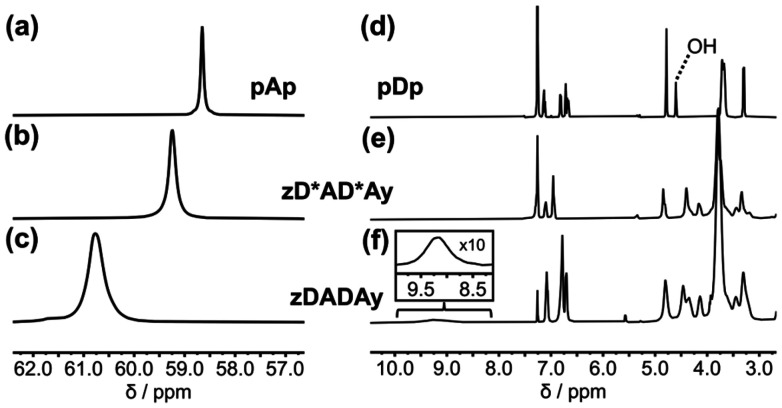
Partial ^31^P NMR spectra (162 MHz, CDCl_3_, 298 K) of 1 mM solutions of (a) pAp, (b) zD*AD*Ay, (c) zDADAy. Partial ^1^H NMR spectra (400 MHz, CDCl_3_, 298 K) of 1 mM solutions of (d) pDp, (e) zD*AD*Ay, (f) zDADAy.

Fig. 5 compares the NMR spectra of zDADAy in deuterochloroform with reference compounds that cannot self-assemble, because they lack complementary recognition units. At a concentration of 1 mM, there is one broad signal at about 61 ppm in the ^31^P NMR spectrum of zDADAy. The corresponding ^31^P NMR signals for zD*AD*Ay and pAp were observed at 59.2 and 58.7 ppm respectively. Formation of a H-bond with a phenol leads to an increase of about 3.0 ppm in the ^31^P NMR chemical shift of a phosphine oxide signal in deuterochloroform (*cf.* the ^31^P NMR data for the AAA·DDD titration in Fig. S23†). The increase in chemical shift observed for zDADAy at a concentration of 1 mM is 1.6 ppm compared with zD*AD*Ay, which is half of the change observed for formation of a fully H-bonded complex (3.0 ppm), suggesting that about 50% of the phosphine oxide groups in zDADAy are H-bonded. The ITC results in Fig. 3(b) indicate that only 30% of the molecules are present as the dimeric complex at this concentration, so the NMR data shows that there is a significant amount of H-bonding in the single-stranded species. This conclusion is supported by the ^1^H NMR spectra in Fig. 5, which show that the signal due to the phenol OH group of zDADAy is observed at 9.2 ppm, compared with 4.6 ppm for pDp. The large difference in chemical shift is characteristic of H-bonding interactions between the phenol and phosphine oxide groups in zDADAy.

Self-assembly of zDADAy was also studied by thermal denaturation experiments. The ^31^P NMR spectrum of a 1 mM solution of zDADAy in 1,1,2,2-tetrachloroethane-*d*_2_ was recorded at different temperatures between 253 and 392 K (see Fig. S24†). At high temperatures, the chemical shift of the ^31^P NMR signal tended towards the value observed for zD*AD*Ay, which is consistent with disruption of phenol–phosphine oxide H-bonding interactions.

### Covalent trapping

**Fig. 6:**
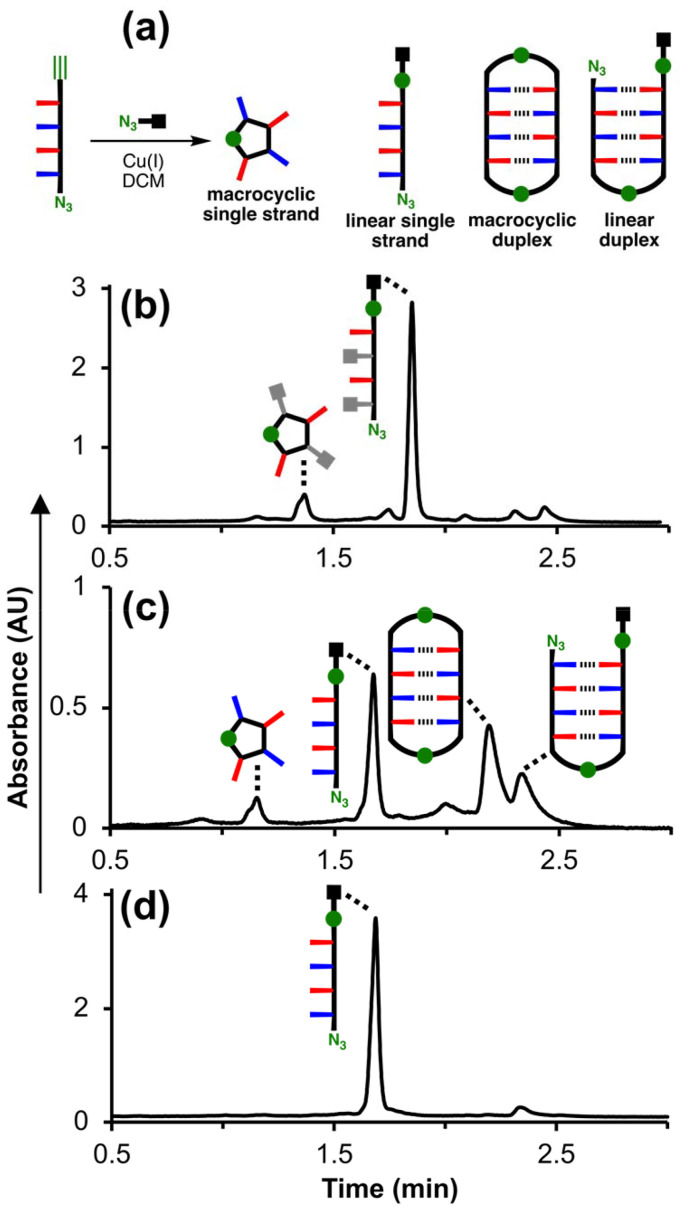
(a) Schematic representation of products formed after CuAAC reaction of zDADAy in the presence of a competing azide (green circles represent triazoles in the products). (b) UPLC trace after reaction of zD*AD*Ay (1 mM), 4-*t*-butylbenzyl azide (1 mM) and Cu(MeCN)_4_PF_6_-TBTA (4 mM) in dichloromethane at room temperature for 48 h. (c) UPLC trace after reaction of zDADAy (1 mM), 4-*t*-butylbenzyl azide (1 mM) and Cu(MeCN)_4_PF_6_-TBTA (4 mM) in dichloromethane at room temperature for 48 h. (d) UPLC trace after reaction of zDADAy (1 mM), 4-*t*-butylbenzyl azide (50 mM) and Cu(MeCN)_4_PF_6_-TBTA (4 mM) in dichloromethane at room temperature for 48 h. *UPLC conditions*: C4 column at 40 °C (254 nm) using a 65–100% gradient of MeCN/formic acid (0.1%) in water/formic acid (0.1%) over 2 minutes, then 100% MeCN/formic acid (0.1%) over 1 minute.

The experiments described above show that the DADA oligomers undergo 1,4-folding at low concentrations but do not provide any information on the structure of the dimeric complex formed at high concentrations, *i.e.* whether it is the duplex or kissing stem-loops dimer. Oligomers zDADAy and zD*AD*Ay were synthesised with terminal azide and alkyne groups, so that CuAAC reactions could be used to covalently trap the supramolecular assemblies present at high concentrations. Fig. 6(a) shows cartoon representations of the products that could be formed if zDADAy is reacted under CuAAC conditions in the presence of a competing azide, which is used to intercept intermolecular reactions that would lead to polymerisation and complicate analysis of the product distribution. Assembly of two zDADAy oligomers as a duplex places the alkyne on the end of one strand in close proximity to the azide on the end of the other strand, so CuAAC reactions at each end of the duplex would give rise to the macrocyclic duplex product. If an intermolecular reaction with the competing azide intercepts one of these reactions, the linear duplex shown in Fig. 6(a) would be formed. The macrocyclic single-strand product would be formed if the alkyne of one strand reacts with the azide of the same strand, which could occur for any of the four structures shown in Fig. 2: the single-strand, the folded single-strand, the duplex or the kissing stem loops dimer. Intermolecular reactions of any of these species with the competing azide would give rise to the linear single strand.

CuAAC trapping experiments were carried out at micromolar concentrations, where zDADAy should exist predominantly in the single-stranded state, and at millimolar concentrations, where the dimeric zDADAy·zDADAy complex should be significantly populated. As a control, CuAAC experiments were carried out on zD*AD*Ay at the same concentrations to establish the product distribution in the absence of H-bonding interactions between the recognition groups.

Fig. 6(b) shows the UPLC trace after the CuAAC reaction of 1 mM zD*AD*Ay in the presence of 1 mM 4-*t*-butylbenzyl azide. The peaks were assigned based on the masses observed in the corresponding mass spectra. The linear single strand was the major product, and a small amount of the macrocyclic single strand was also formed. In contrast, when 1 mM zDADAy was reacted under the same conditions, the UPLC trace showed that the macrocyclic duplex and linear duplex were the major products (Fig. 6(c)). Comparison of the trapping experiments for zD*AD*Ay and zDADAy indicates that the duplex products in Fig. 6(c) are formed due to H-bonding interactions involving the phenol recognition units. The proportion of macrocyclic single-strand product is similar in the two experiments, which indicates that H-bonding does not promote this product. Fig. 6(d) shows the UPLC trace after CuAAC reaction of 1 mM zDADAy in the presence of a large excess of 4-*t*-butylbenzyl azide (50 mM). In this case, intermolecular interactions with the competing azide dominate, and the duplex products were almost entirely eliminated to yield the linear single strand as the major product. This result further supports the conclusion that formation of the macrocyclic duplex and linear duplex species in Fig. 6(c) arises from intramolecular reactions within the zDADAy·zDADAy duplex.

**Fig. 7:**
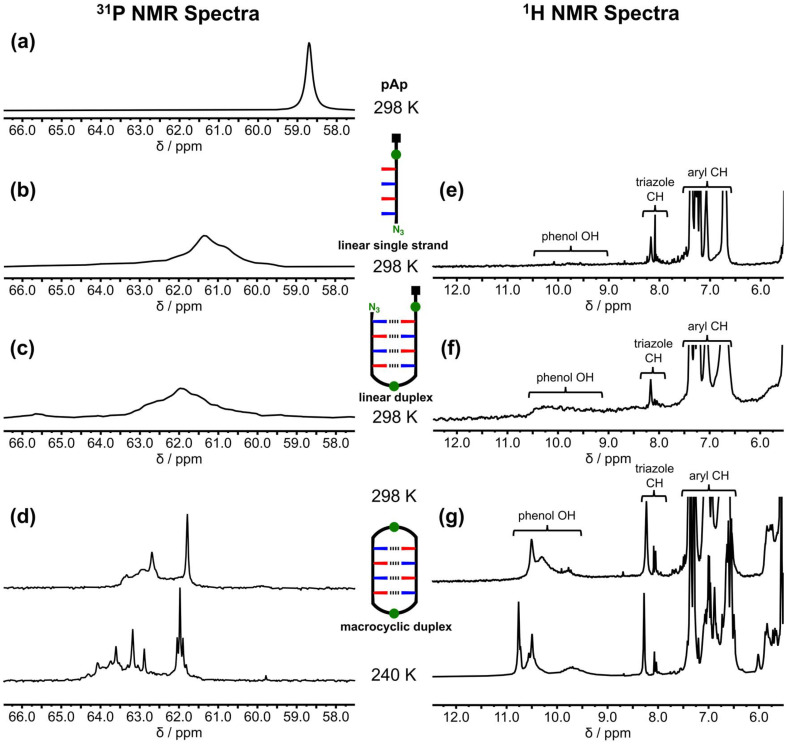
NMR spectra of isolated products from the covalent CuAAC trapping reaction of zDADAy. Partial ^31^P NMR spectra (162 MHz, dichloromethane-*d*_2_) of (a) phosphine oxide monomer pAp at 298 K, (b) linear single strand at 298 K, (c) linear duplex at 298 K, (d) macrocyclic duplex at 298 and 240 K. Partial ^1^H NMR spectra (400 MHz, dichloromethane-*d*_2_) of (e) linear single strand at 298 K, (f) linear duplex at 298 K, (g) macrocyclic duplex at 298 and 240 K. All spectra were recorded at approximately 1 mM concentration. Line broadening of 30 Hz was applied to the ^31^P NMR spectra in (a), (b) and (c), and 2 Hz to the spectra in (d).

The three major products from the zDADAy trapping reaction in Fig. 6(c) were isolated by preparative HPLC (see Fig. S27†). ^31^P and ^1^H NMR spectroscopy was used to probe the phenol·phosphine oxide H-bonding interactions present within these species in deuterodichloromethane. Fig. 7(b) shows the ^31^P NMR spectrum of the linear single strand. There is a broad peak at about 61 ppm, which is similar to the chemical shift observed for the starting material zDADAy in deuterochloroform (Fig. 5(c)). This chemical shift is downfield of the free phosphine oxide chemical shift measured for pAp (Fig. 7(a)) and indicates that the phosphine oxide groups are partially H-bonded. In the ^1^H NMR spectrum of the linear single-stranded product, the signal due to the phenol OH proton is present as a very broad peak between 9.0 and 10.5 ppm, which is consistent with partially H-bonded phenol groups (Fig. 7(e)).

For the linear duplex, there is a broad peak at about 62 ppm in the ^31^P NMR spectrum, which is 1 ppm downfield of the corresponding signal for the linear single-stranded product, suggesting that the phosphine oxide groups are H-bonded to a greater extent in the duplex (Fig. 7(c)). In the ^1^H NMR spectrum of the linear duplex product, the signal due to the phenol OH is more clearly visible as a broad peak between 9.0 and 10.5 ppm (Fig. 7(f)).

The NMR spectrum of the macrocyclic duplex product is quite different. In the room temperature ^31^P NMR spectrum, there is a sharp signal at 61.8 ppm and a broad peak around 63 ppm (Fig. 7(d)). When the spectrum was recorded at 240 K, the resolution of the signals increased. There are three sharp signals that resemble a 1 : 2 : 1 triplet at 62 ppm, three more sharp signals that resemble 1 : 2 : 1 triplet between 63 and 64 ppm, and a broader set of signals spread out between 62.5 and 64.5 ppm. In the ^1^H NMR spectrum of the macrocyclic duplex product recorded at 240 K (Fig. 7(g)), the signals due to the phenol OH groups appear as two sharp signals at 10.5 and 10.8 ppm and a broad signal at 9.7 ppm. Both the ^31^P and ^1^H NMR spectra indicate that there is an increase in the extent to which the recognition units are H-bonded in the macrocyclic duplex compared with the other products. Although the increased resolution suggests that the conformation of the macrocyclic duplex is more well-defined, the increase in the complexity of the spectra implies that multiple conformers are populated. For example, there are at least seven different ^31^P signals for a compound that contains four phosphorus atoms, which suggests slow exchange between conformers, for example involving rotamers around the exocyclic carbon–nitrogen bonds of the triazines. The NMR results show that formation of triazoles between the terminal azide and alkyne units of the zDADAy·zDADAy duplex promotes the formation of H-bonded base-pairs, which validates the CuAAC approach as a method for trapping these supramolecular assemblies. It is possible that interactions with the copper complex could affect the distribution of species obtained in the trapping reaction, but it is clear that the H-bonding interactions that lead to formation of the duplex are intact in the reaction medium.

**Fig. 8:**
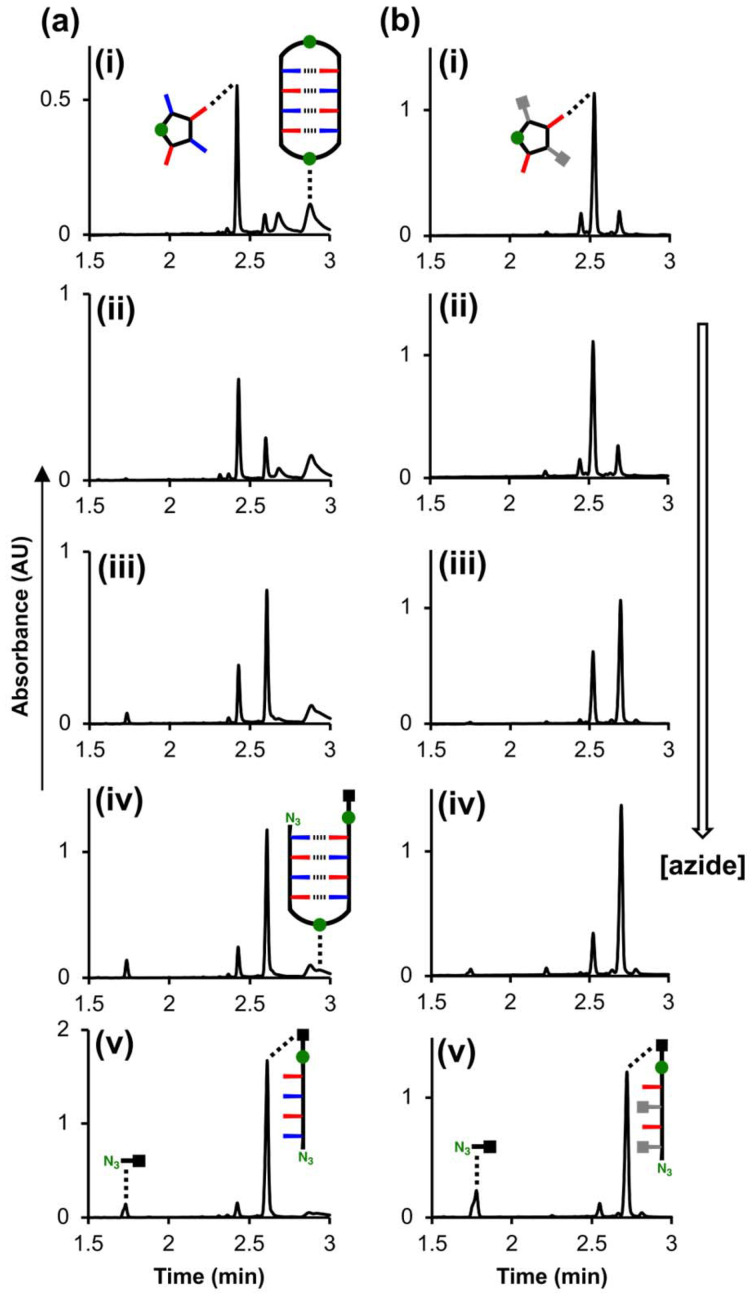
(a) UPLC traces of product mixtures after CuAAC reaction of zDADAy in the presence of competing azide (green circles represent triazoles). Reactions of zDADAy (25 μM), 4-*t*-butylbenzyl azide ((i) 25 μM, (ii) 100 μM, (iii) 450 μM, (iv) 1 mM, (v) 2.5 mM) and Cu(MeCN)_4_PF_6_-TBTA (0.1 mM) were carried out in DCM at r.t. for 48 h. (b) UPLC traces of product mixtures after CuAAC reactions of zD*AD*Ay in the presence of competing azide (green circles represent triazoles). Reactions of zD*AD*Ay (25 μM), 4-*t*-butylbenzyl azide ((i) 25 μM, (ii) 100 μM, (iii) 450 μM, (iv) 1 mM, (v) 2.5 mM) and Cu(MeCN)_4_PF_6_-TBTA (0.1 mM) were carried out in DCM at r.t. for 48 h. *UPLC conditions*: C4 column at 40 °C (254 nm) using a 5–100% gradient of MeCN/formic acid (0.1%) in water/formic acid (0.1%) over 2 minutes, then 100% MeCN/formic acid (0.1%) over 1 minute.

The trapping experiments were then repeated at much lower concentrations to reduce the proportion of duplex present. Fig. 8(a) shows the UPLC traces of product mixtures obtained after CuAAC reactions of 25 μM zDADAy in the presence of different concentrations of 4-*t*-butylbenzyl azide. At low concentrations of competing azide, the macrocyclic single-stranded species was the major product, and some macrocyclic duplex was observed. As the concentration of competing azide was increased, both macrocyclic species were intercepted, and the amount of linear single-stranded product increased. Fig. 8(b) shows the corresponding UPLC traces of the product mixtures obtained after CuAAC reactions of 25 μM zD*AD*Ay under the same conditions. At low concentrations of competing azide, the macrocyclic single-stranded species was again the major product, and at high concentrations of competing azide, the linear single-stranded species was the major product. No duplex products were observed in this case, because zD*AD*Ay cannot form H-bonded base-pairs.

Comparison of the results for zDADAy and zD*AD*Ay in Fig. 8 suggests that the yield of the macrocyclic single-stranded product does not depend on the presence of H-bonding interactions between sequence-complementary chain ends. The effect of the competing azide on the product distribution can be used to determine the effective molarity (EM) for the intramolecular macrocyclisation reactions observed for the two single-stranded oligomers. The rates of the intramolecular reaction to form the macrocyclic single-stranded product and the intermolecular reaction to form the linear single-stranded product can be written as eqn (1) and (2), where *k*_1_ and *k*_2_ represent first and second order rate constants respectively.

1 Rate_intra_ = *k*_1_[oligomer] = *k*_2_EM[oligomer]

2 Rate_inter_ = *k*_2_[oligomer][4-*t*-butylbenzyl azide]

Although the nature of the azide groups differs in the two reactions, control experiments showed that the reactivity of the azides is identical (see ESI†), so the same second order rate constant can be used in both equations. Assuming that extinction coefficients of the two products are similar, the integrals of the peaks in the UPLC traces corresponding to the macrocyclic single-stranded product (*A*_intra_) and the linear single-stranded product (*A*_inter_) can be used in eqn (3) to estimate the value of EM.3
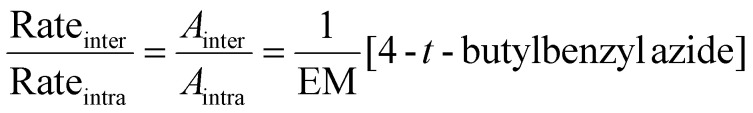


**Fig. 9:**
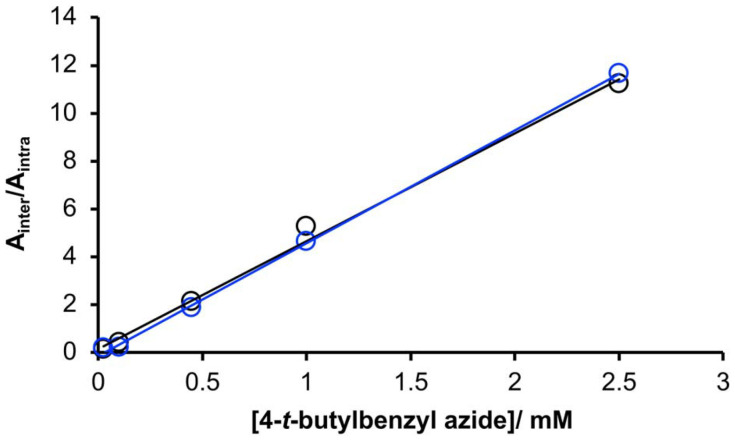
Product distribution for the CuAAC reaction of zDADAy (black) and zD*AD*Ay (blue) in the presence of different concentrations of 4-*t*-butylbenzyl azide (Fig. 8), plotted as the ratios of the areas of the UPLC peaks assigned to the linear single stranded products (*A*_inter_) compared with the areas of the UPLC peaks assigned to the macrocyclic linear single stranded products (*A*_intra_). Lines represent the best fit of the data to eqn (3) (EM = 0.2 mM).

Fig. 9 shows the product distributions plotted as a function of the concentration of 4-*t*-butylbenzyl azide for zDADAy and zD*AD*Ay. The value of EM (0.2 mM) was identical for zDADAy and zD*AD*Ay, so the intramolecular H-bonding interactions in zDADAy have no effect on the probability of reaction between the terminal alkyne and azide groups. This result might suggest that single-stranded zDADAy does not fold, but this conclusion would contradict the ITC experiments described above. More likely, the spatial arrangement of the terminal alkyne and azide groups in the folded single-stranded state does not promote the CuAAC in the same manner as the duplex. Thus the CuAAC trapping experiment, whilst an effective tool for investigating the formation of H-bonded duplexes, does not provide any insight into 1,4-folding in the REMO architecture.

## Conclusions

Competition between intramolecular and intermolecular processes is a general phenomenon that governs self-assembly and folding of macromolecules. Recognition-encoded melamine oligomers (REMO), which are composed of a backbone of repeating triazine-piperidine units and side-chains bearing complementary phenol (D) and phosphine oxide (A) recognition units, form sequence-selective H-bonded duplexes. The major pathway that competes with duplex formation in mixed sequence oligomers is intramolecular folding due to H-bonding interactions between two recognition units on the same strand. We have previously shown that REMO are sufficiently rigid to prevent 1,2- and 1,3-folding, and here 1,4-folding was investigated using 4-mers with the sequence DADA. Two oligomers, pDADAp and zDADAy, were synthesised *via* automated solid phase synthesis, one with terminal piperidine (p) units, and one with terminal alkyne (y) and azide (z) units for use in covalent trapping experiments using CuAAC reactions.

An ITC dilution experiment gave a value of 300 M^−1^ for the pDADAp·pDADAp self-association constant in chloroform, which is significantly lower than the value measured for an AAA·DDD duplex that forms three intermolecular H-bonds. This result suggests that there is an intramolecular 1,4-interaction between the two recognition groups on the end of the oligomer, so when two folded pDADAp oligomers form a duplex with four intermolecular H-bonds, the gain in the number of H-bonds is only two.

It is possible that the intramolecular H-bonds that lead to 1,4-folding persist in the dimeric pDADAp·pDADAp complex characterised by ITC. In order to rule out this kissing stem-loops structure, CuAAC covalent trapping experiments were carried out using zDADAy and zD*AD*Ay, an analogue where self-assembly due to H-bonding cannot occur because the phenol units are acetylated. The sequence of zDADAy dictates that the terminal azide and alkyne groups on two different strands should be in close proximity in the duplex structure, and that the terminal azide and alkyne groups on the same strand should be in close proximity in the kissing stem loops structure. When the CuAAC reaction was carried out on zDADAy at millimolar concentrations in dichloromethane, the macrocyclic duplex was the major product observed. For zD*AD*Ay under the same conditions only single-stranded products were observed, which confirms that H-bonding interactions between the phenol and phosphine oxide recognition units are responsible for self-assembly of the zDADAy·zDADAy duplex under these conditions.

When the CuAAC covalent trapping reaction was repeated with zDADAy at micromolar concentrations, where the dimeric complex is not populated, the single-stranded macrocycle was the major product, which is consistent with the 1,4-folded single-strand structure. However, similar results were observed for zD*AD*Ay. This result implies that folding of zDADAy does not bring the terminal alkyne and azide groups into sufficiently close proximity to promote the CuAAC reaction compared with zD*AD*Ay, which does not fold.

Overall these experiments show that 1,4-folding competes with duplex formation in REMO. Although the intramolecular H-bond between the terminal phenol and phosphine oxide recognition units in the folded single-stranded state reduces the stability of the duplex, the duplex is the major species present at millimolar concentrations in organic solvents. It is not possible to rule out some population of a kissing stem-loops structures in the zDADAy·zDADAy complex, but no evidence for this structure was found, and the covalent trapping experiments show that the fully assembled duplex is the major species.

## Author contributions

The manuscript was written through contributions of all authors.

## Conflicts of interest

There are no conflicts to declare.

## Supplementary Material

OB-023-D5OB00769K-s001

## Data Availability

All supporting data is provided in the ESI.†
