# Supplementary material for: Supramolecular assembly properties of a mixed-sequence recognition-encoded melamine oligomer
Source: Org Biomol Chem. 2025 Jul 4;23(29):6948–56. doi: 10.1039/d5ob00769k (PMC12227063; doi:10.1039/d5ob00769k)
Supplement: OB-023-D5OB00769K-s001 [file OB-023-D5OB00769K-s001.pdf]

Supplementary Information (SI) for Organic & Biomolecular Chemistry.
This journal is © The Royal Society of Chemistry 2025

Supramolecular assembly properties of a mixed-sequence recognition-encoded melamine oligomer

Mohit Dhiman,[1] Joseph T. Smith,[1] Christopher A. Hunter [1,*]

[1]Yusuf Hamied Department of Chemistry, University of Cambridge, Cambridge CB2 1EW, U.K.

*Email: herchelsmith.orgchem@ch.cam.ac.uk

Supplementary Information

1. **General Experimental Details** S2
2. **Synthesis of Building Blocks** S3
3. **Functionalisation of Wang Resin** S5
4. **Protocols for Automated Solid-Phase Synthesis** S6
5. **Oligomer Synthesis** S8
6. **ITC Dilution of pDADAp** S29
7. **^{31}P NMR Experiments** S31
8. **CuAAC Duplex Trapping Experiments** S33

1. General Experimental Details

All reagents and materials used in the syntheses described were bought from commercial sources and used without prior purification. Dry solvents were obtained from a Grubbs PS-MD-5 solvent purification system and used with no further degassing. Thin layer chromatography (TLC) was carried out using silica gel 60F (Merck) on glass plates. LCMS analyses of samples were performed using a Waters Acquity H-class UPLC coupled with a single quadrupole Waters SQD2. Two different UPLC columns were used: an Acquity UPLC CSH C18 Column (130 Å, 1.7 µm, 2.1 mm x 50 mm), and an Acquity UPLC PRM PR BEH C4 Column (300 Å, 1.7 µm, 2.1 mm x 50 mm).

Purification of compounds by silica column chromatography was performed using an automated system (Combiflash® Rf+ or Combiflash® Rf+ Lumen) with pre-packaged silica cartridges (25 µm or 50 µm PuriFlash® columns). All NMR spectra were recorded using a Bruker 500 MHz Avance III Smart Probe Spectrometer, a Bruker 400 MHz Avance III HD Spectrometer, a Bruker 400 MHz Avance III HD Smart Probe Spectrometer, or a Bruker 400 MHz Neo Prodigy Spectrometer at 298 ± 0.1 K. The residual ^{1}H form of the solvent was used as the internal standard for referencing. In chloroform-*d*, the ^{1}H spectra were referenced to δ 7.26 ppm and ^{13}C spectra referenced to δ 77.16 ppm. In dichloromethane-d_2, the ^{1}H spectra were referenced to δ 5.31 ppm. Chemical shifts (δ) are quoted in ppm and coupling constants (*J*) quoted in Hz. Splitting patterns are reported as: s (singlet), bs (broad singlet), d (doublet), t (triplet), q (quartet) and m (multiplet). FT-IR spectra were collected with an ALPHA FT-IR Spectrometer from Bruker. HRMS spectra were recorded using a Waters SQD2 with Waters H-Class UPLC, equipped with a Waters Acquity UPLC BEH C18 Column (130 Å, 1.7µm, 2.1 mm x 50 mm).

Analytical reverse-phase HPLC was performed on an Agilent HP-1100 Series HPLC system. Preparative reverse-phase HPLC was performed on an Agilent HP-1100 Series preparative HPLC system.

UV-vis spectra were collected on an Agilent Cary 60 UV-vis spectrophotometer controlled by Cary WinUV software.

2. Synthesis of Building Blocks

Synthesis of 1, 3, 4 and 5

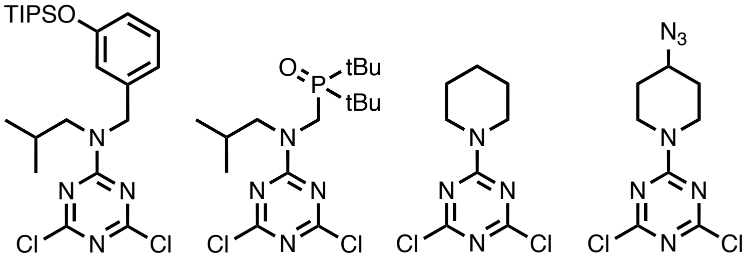

1 **3** **4** **5**

Compounds **1**, **3**, **4**, and **5** were synthesised according to the literature procedures found at:

M. Dhiman, R. Cons, O. N. Evans, J. T. Smith, C. J. Anderson, R. Cabot, D. O. Soloviev, and C. A. Hunter, *J. Am. Chem. Soc.*, 2024, **146**, 9236–9334.

Synthesis of 2

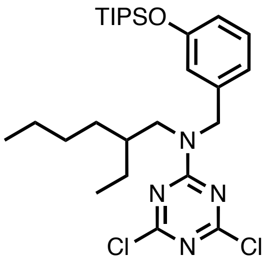

2

Compound **2** was synthesised according to the literature procedure found at:

M. Dhiman, R. Cabot and C. A. Hunter, *Chem. Sci.*, 2024, **15**, 5957–5963.

Synthesis of 6 and 7

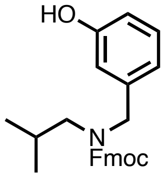

6

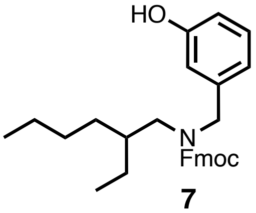

7

Compounds **6** and **7** were synthesised according to the literature procedures found at:

M. Dhiman, R. Cabot and C. A. Hunter, *Chem. Sci.*, 2024, **15**, 5957–5963.

3. Functionalisation of Wang Resin

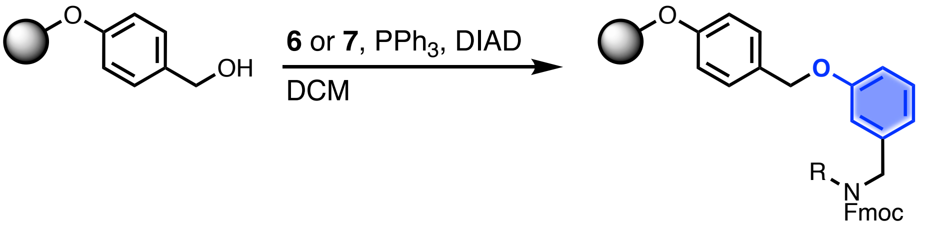

TentaGel Wang Resin (90 μm mesh) was swollen in dry DCM for 30 min before a solution of **6** or **7** and triphenylphosphine in dry DCM was added to the resin. A solution of diisopropyl azodicarboxylate was diluted 5-fold in dry DCM then added dropwise to the resin. The resin was agitated at r.t. overnight, and then washed alternatingly with DCM (5x) and DMF (5x) to yield the resin.

Quantification of resin loading: Functionalised Wang resin was treated with a solution of DBU in DMF (2 mL, 2 vol. %) and agitated for 30 mins. 1 mL of the solution was removed from the resin and diluted with 4 mL acetonitrile. A 1 mL aliquot of the resultant solution was taken and diluted to 12.5 mL with acetonitrile. The absorbance of the DBU-fulvene adduct (λ = 304 nm, ε = 9254 M^{-1} cm^{-1}) was measured to estimate the resin loading.

4. Protocols for Automated Solid-Phase Synthesis

General methods using CEM Liberty Blue Automated Synthesiser: Heated automated Solid-Phase Synthesis (SPS) was performed on a CEM Liberty Blue automated synthesiser on a 50 µmol scale. Solutions of piperazine (0.7 M) in DMF, **1** (0.125 M) in DMF, **2** (0.125 M) in DMF, **3** (0.125 M) in DMF, **4** (0.125 M) in DMF, **5** (0.125 M) in DMF and DIPEA (0.5 M) in DMF were prepared for coupling. General synthetic protocols performed were:

Fmoc deprotection: The loaded Wang resin was agitated in a solution of piperazine in DMF (7 mL, 0.7 M, 2 x 10 min). The deprotection solution was then drained and the resin was washed with DMF (4 x 5 mL).

Coupling cycle: The resin-bound oligomer was first agitated in a solution of **1**, **2**, **3**, **4** or **5** (0.1 M, 10 eq.) and DIPEA (0.1 M, 10 eq.) in DMF (5 mL) for 10 or 15 mins at 90 °C. The 1st coupling solution was drained, and the resin was washed with DMF (4 x 5 mL). The resin-bound oligomer was then agitated in a solution of piperazine (5 mL, 0.7 M) in DMF for 10 or 15 min at 90 °C. The 2nd coupling solution was drained, and the resin was washed with DMF (4 x 5 mL).

4-Ethynylpiperidine/piperidine coupling (manual MW-assisted SPS): The resin-bound oligomer was swollen in DMF for 15 min before being agitated in a solution of 4-ethynylpiperidine trifluoroacetate (0.1 M) or piperidine (0.1 M) and DIPEA (1 M) in DMF (5 mL) for 30 mins at 90 °C. The coupling solution was drained, and the resin washed with DMF (4 x 5 mL).

TIPS deprotection: The resin-bound oligomer was swollen in THF for 15 min before being agitated in a solution of TBAF (5 mL, 1M in THF) at r.t. for 1h. The solution was drained, and the resin washed with MeOH and THF alternatingly (4 x 5 mL) before being subjected to another deprotection cycle.

Resin Cleavage: The resin was agitated in a mixture of TFA:TIS:DCM (90:5:5 v/v/v) at r.t. for 2 h. The resin was filtered and washed with DCM (5 x 5 mL) and then subjected to another cleavage cycle. The combined filtrates from both cleavage cycles were concentrated under N_2 flow before drying *in vacuo*.

HPLC Purification: Analytical reverse-phase HPLC was performed on an Agilent HP-1100 Series HPLC system composed of a high-pressure binary pump, an autosampler with injector programming capabilities and a diode array detector with a semimicro flow cell (6 mm path length, 5 µL volume). UV/vis absorption was measured at 240 nm and 280 nm (8 nm bandwidth). A Waters XBridge BEH C8 (130 Å, 2.5 µm), 6 x 75 mm column was employed at a flow rate of 0.7 mL/min.

Preparative reverse-phase HPLC was performed on an Agilent HP-1100 Series preparative HPLC system composed of a high-pressure mixing binary pump, dual injector autosampler loops (50 µL/5 mL loops), a variable UV/vis detector (190 nm to 600 nm) and an automatic fraction collector. UV/vis absorption was measured at 240 nm and 280 nm (8 nm bandwidth) and fractions were set to collect automatically based on peak threshold. A Waters XBridge BEH C8 OBD Prep (130 Å, 5 µm), 19 x 250 mm column was employed at a flow rate of 10 mL/min.

Both instruments utilised a mobile phase composed of 95:5 water (HPLC grade):MeCN (HPLC grade) as solvent A, and THF (HPLC grade) as solvent B operating using a linear gradient.

5. Oligomer Synthesis

Synthesis of pDADAp

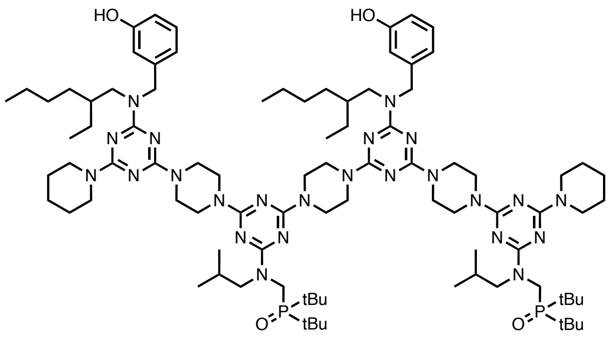

TentaGel Wang resin was loaded with **7** in accordance with the general methods. The loaded resin (50 µmol) was subjected to SPS on a CEM Liberty Blue automated synthesiser, followed by coupling with 4-ethynylpiperidine via manual SPS. TIPS deprotection followed by resin cleavage afforded the crude oligomer. The crude oligomer was purified via preparative HPLC (65% B over 40 mins) to yield the oligomer **pDADAp** (33 mg, 58% based on the initial resin loading) as a white solid.

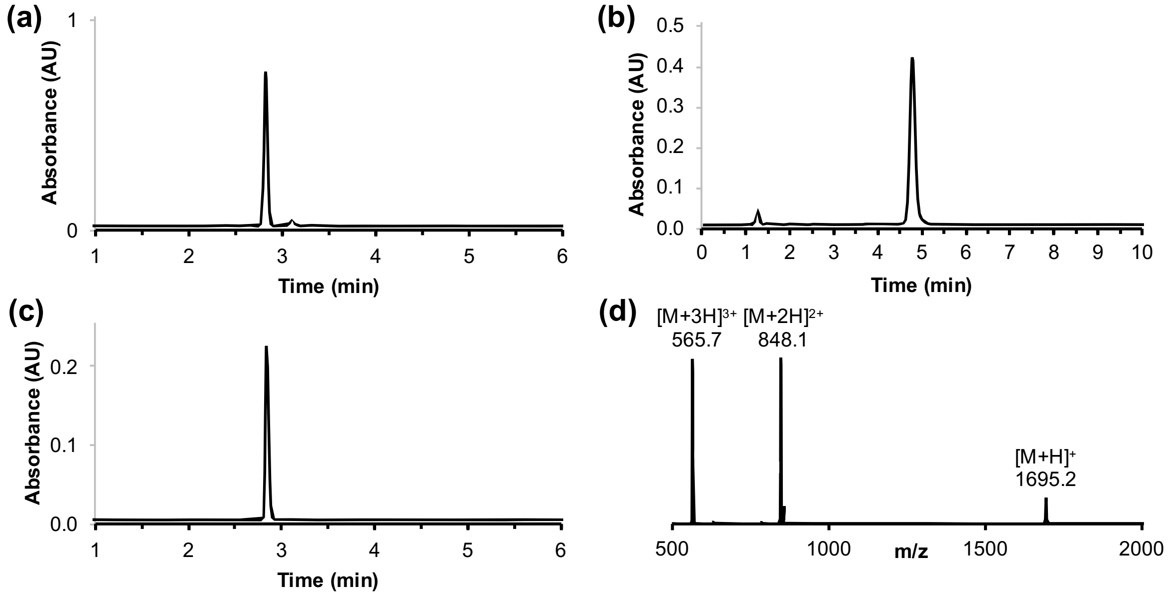

Figure S1: (a) Crude UPLC trace of **pDADAp**. **(b)** Crude analytical HPLC trace of **pDADAp**. **(c)** UPLC trace after purification. **(d)** ESI-MS of **pDADAp**. Calculated mass (ESI^+): 1695.2 $[M+H]^+$, 848.1 $[M+2H]^{2+}$, 565.7 $[M+3H]^{3+}$. *UPLC Conditions:* C4 column at 40 °C using a 30-100% gradient of THF/formic acid (0.1%) in water/formic acid (0.1%) over 4 minutes, then 100% THF/formic acid (0.1%) over 2 minutes.

^{1}H NMR (400 MHz, chloroform-d): δ_H 7.08 (m, 2H, aryl-C$\underline{H}$), 6.79-6.67 (m, 6H, aryl-C$\underline{H}$), 4.89-4.69 (m, 4H, -NC$\underline{H}_2$C$_6$H$_4$OH), 4.49-4.37 (m, 4H, -NC$\underline{H}_2$PO^{t}Bu$_2$), 3.85-3.21 (m, 40H, -NC$\underline{H}_2$- region), 2.24-2.14 (m, 2H), 1.84-1.74 (m, 2H), 1.69-1.47 (m, 28H, -C$\underline{H}_2$-), 1.27 (m, 36H, ^{t}Bu region), 0.89-0.79 (m, 24H, -C$\underline{H}_3$);

^{31}P NMR (162 MHz, chloroform-d): δ_P 60.60, 59.88;

HRMS (ES+): calculated for $C_{90}H_{151}N_{24}O_4P_2$ 1694.1825 [M+H]$^+$, found 1694.1826 [M+H]$^+$.

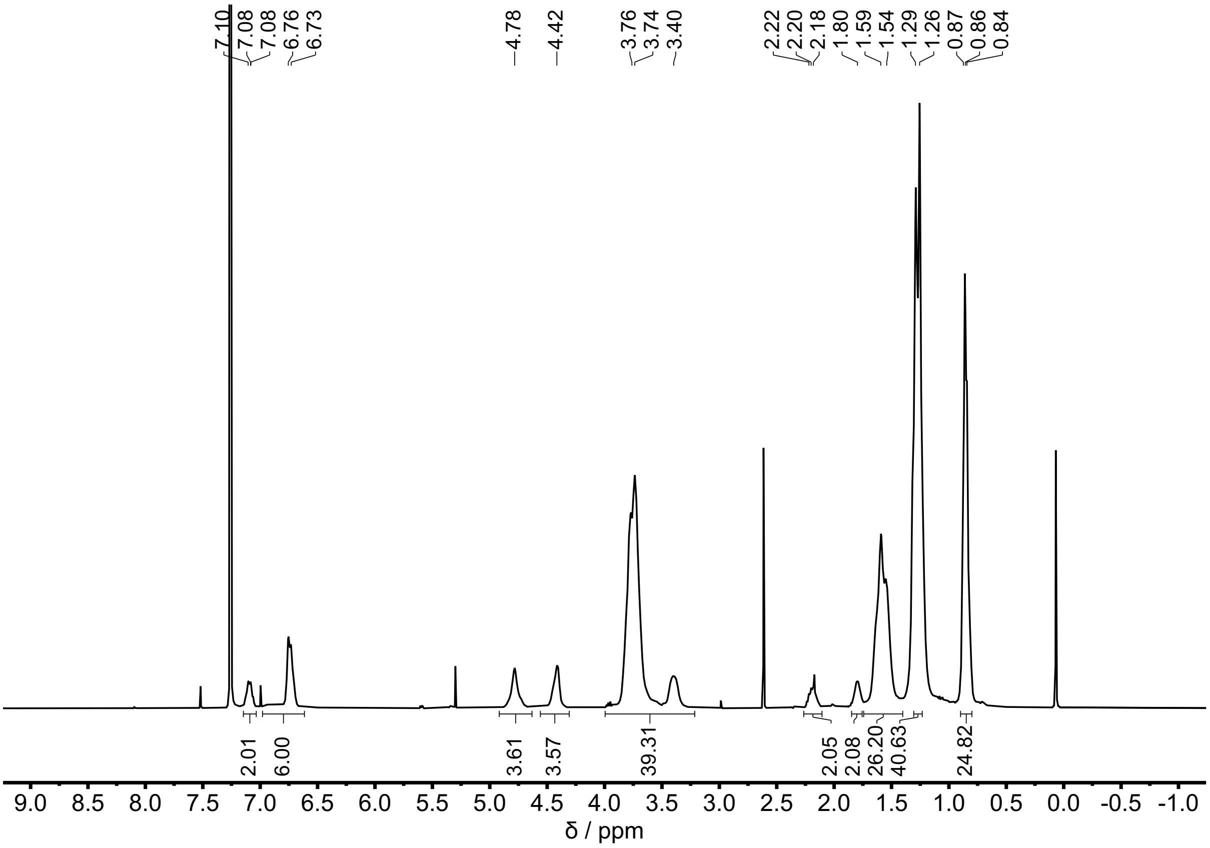

Figure S2: ^{1}H NMR (400 MHz, chloroform-d) spectrum of **pDADAp**.

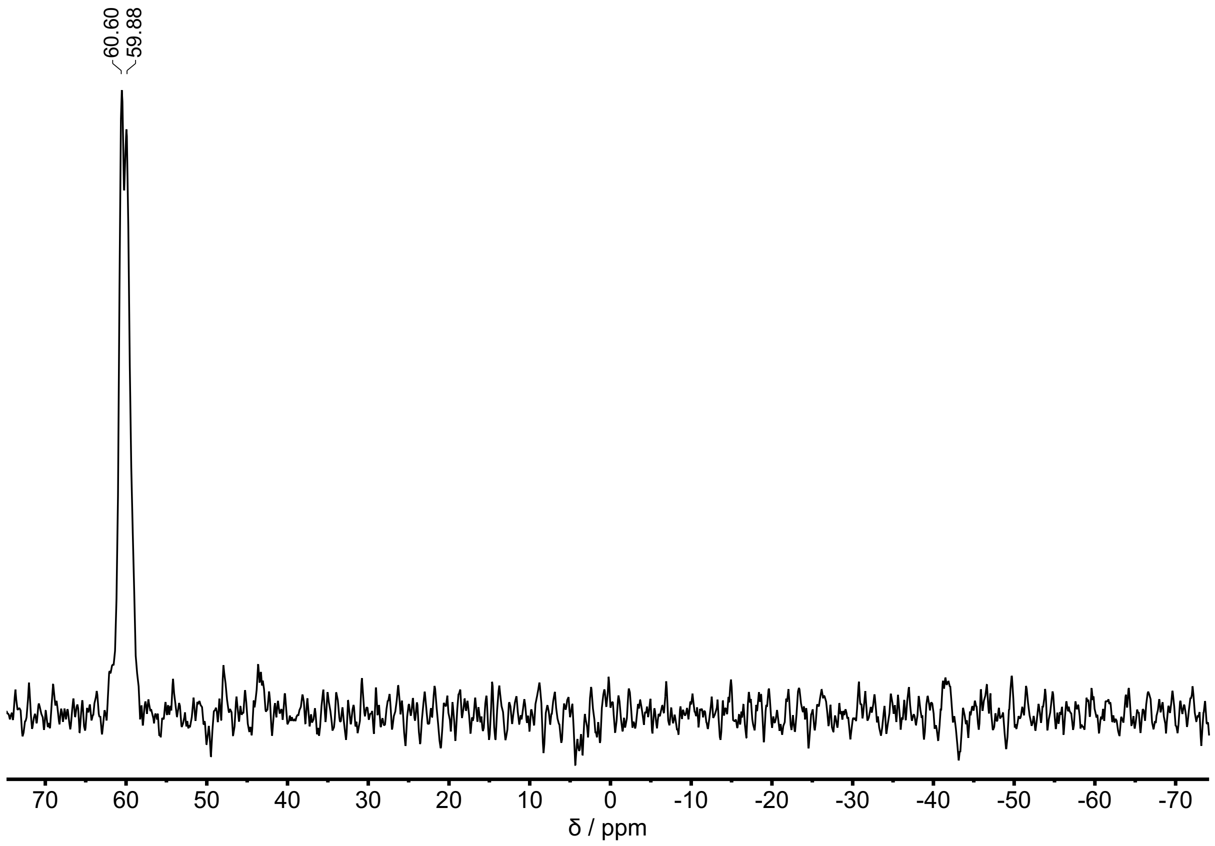

Figure S3: ^{31}P NMR (162 MHz, chloroform-d) spectrum of **pDADAp**.

Synthesis of zDADAy

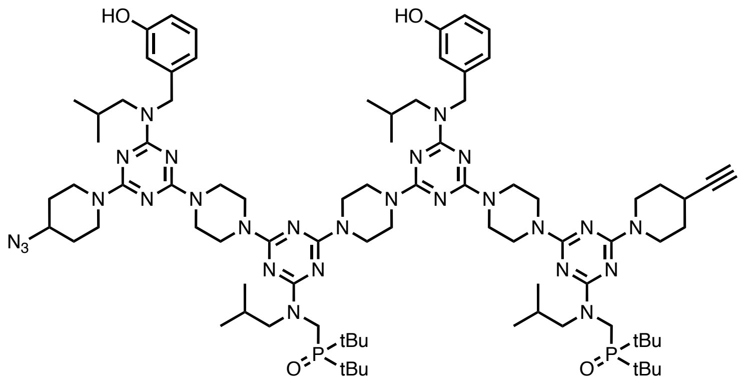

TentaGel Wang resin was loaded with **6** in accordance with the general methods. The loaded resin (50 µmol) was subjected to SPS on a CEM Liberty Blue automated synthesiser, followed by coupling with 4-ethynylpiperidine via manual SPS. TIPS deprotection followed by resin cleavage afforded the crude oligomer. The crude oligomer was purified via preparative HPLC (60% B over 40 mins) to yield the oligomer **zDADAy** (27 mg, 49% based on the initial resin loading) as a white solid.

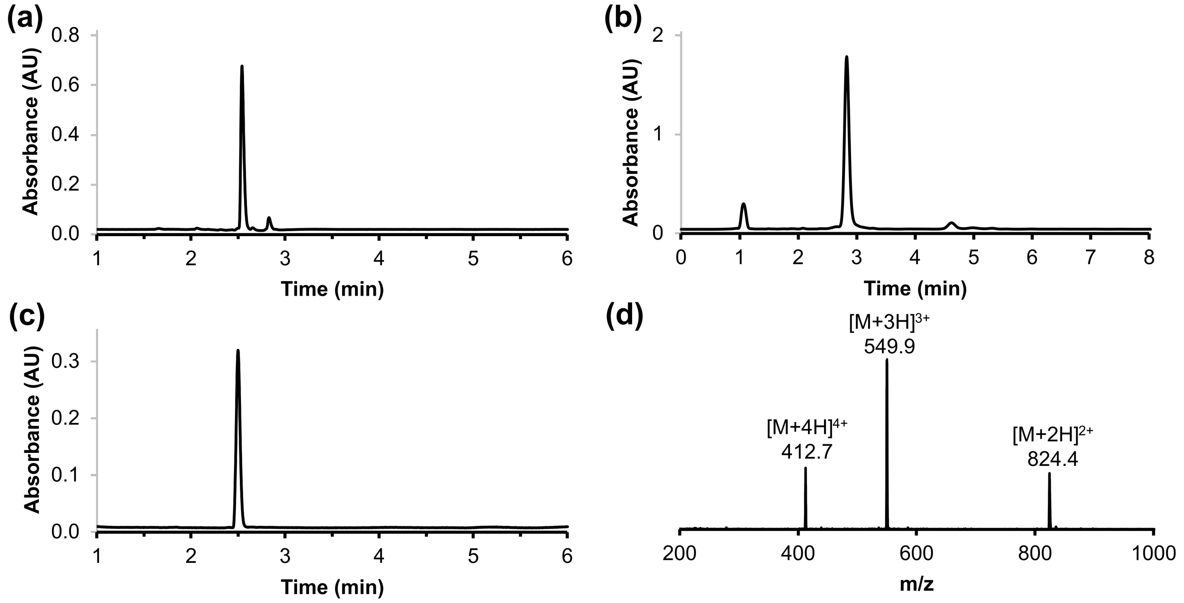

Figure S4: (a) Crude UPLC trace of **zDADAy**. **(b)** Crude analytical HPLC trace of **zDADAy**. **(c)** UPLC trace after purification. **(d)** ESI-MS of **zDADAy**. Calculated Mass: 824.5 $[M+2H]^{2+}$, 550.0 $[M+3H]^{3+}$, 412.8 $[M+4H]^{4+}$. *UPLC Conditions:* C4 column at 40 °C using a 30-100% gradient of THF/formic acid (0.1%) in water/formic acid (0.1%) over 4 minutes, then 100% THF/formic acid (0.1%) over 2 minutes.

^{1}H NMR (400 MHz, chloroform-d): δ_H 9.17 (br s, 2H), 7.08 (t, J = 7.8 Hz, 2H), 6.88-6.74 (m, 4H), 6.74-6.67 (m, 2H), 4.83-4.79 (m, 4H), 4.52-4.26 (m, 6H), 4.17-4.11 (m, 2H), 3.95-3.53 (m, 29H), 3.51-3.39 (m, 2H), 3.37-3.17 (m, 6H), 2.61 (s, 1H), 2.25-2.15 (m, 2H), 2.13-2.05 (m, 3H), 1.98-1.73 (m, 4H), 1.71-1.46 (m, 4H), 1.30 (d, $^{3}J_{PH}$ = 12.6 Hz, 36H), 0.91-0.84 (m, 24H);

^{31}P NMR (162 MHz, chloroform-d): δ_P 61.32;

HRMS (ES+): calculated for $C_{84}H_{133}N_{27}O_4P_2$ 1648.0610 [M+H]$^+$, found 1648.0614 [M+H]$^+$.

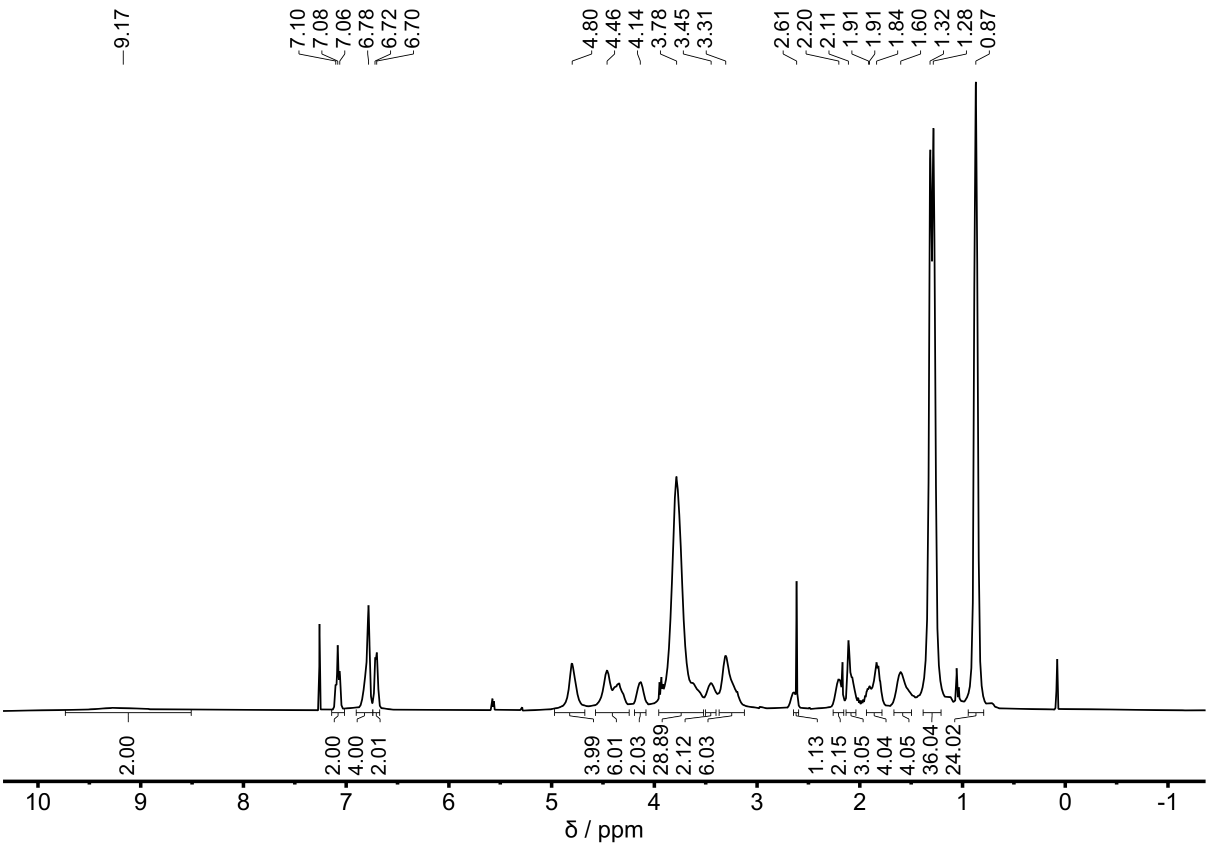

Figure S5: ^{1}H NMR (400 MHz, chloroform-d) spectrum of **zDADAy**.

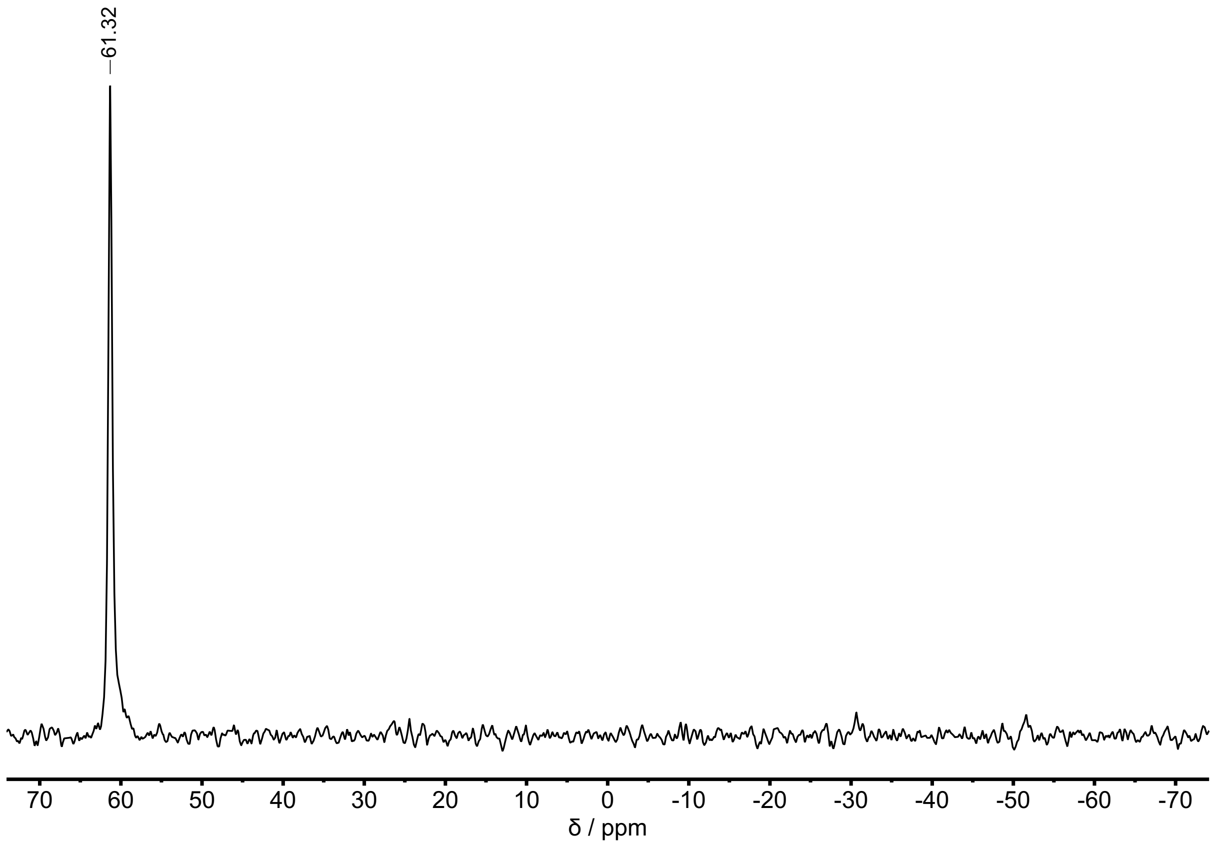

Figure S6: ^{31}P NMR (162 MHz, chloroform-d) spectrum of **zDADAy**.

Synthesis of zD*AD*Ay

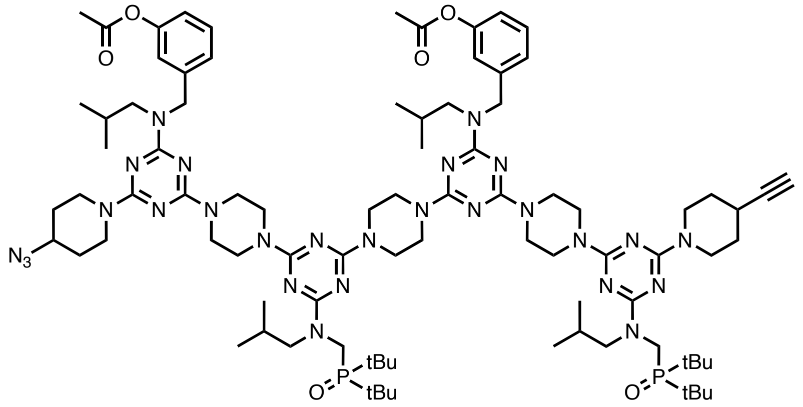

A mixture of **zDADAy** (6 mg, 0.004 mmol), glacial acetic acid (2 mg, 0.04 mmol), EDC (7 mg, 0.04 mmol) and DMAP (5 mg, 0.04 mmol) in dry DCM (3 mL) was stirred at r.t. overnight. The mixture was extracted with EtOAc (3x) and washed with 1 M HCl (2x), H_2O and brine. The organic phase was dried over $MgSO_4$ and the solvent was removed *in vacuo* to yield the crude product. The crude was purified by flash chromatography (SiO_2, 0-10% gradient of methanol in DCM) to yield the pure product **zD*AD*Ay** (5.5 mg, 0.003 mmol, 87%) as a white solid.

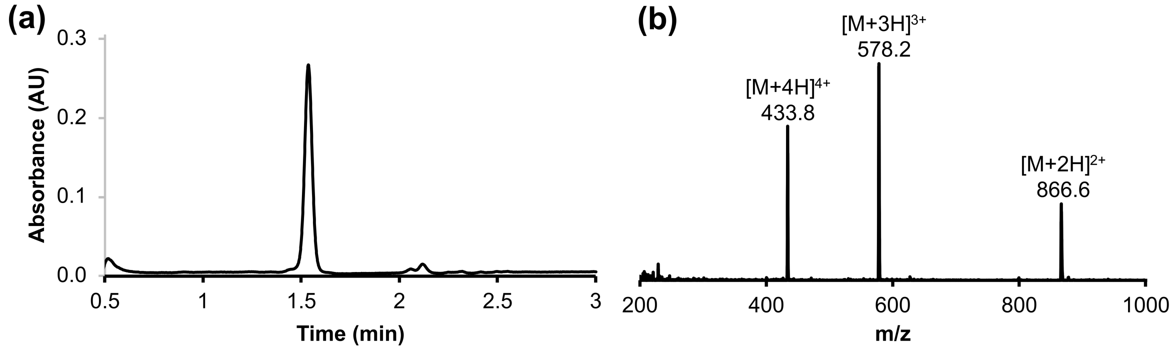

Figure S7: (a) UPLC trace of **zD*AD*Ay**. **(b)** ESI-MS of **D*AD*A**. Calculated mass: 866.5 $[M+2H]^{2+}$, 578.0 $[M+3H]^{3+}$, 433.8 $[M+4H]^{4+}$. *UPLC Conditions:* C4 column at 40 °C using a 65-100% gradient of MeCN/formic acid (0.1%) in water/formic acid (0.1%) over 2 minutes, then 100% MeCN/formic acid (0.1%) over 1 minute.

^{1}H NMR (400 MHz, chloroform-d): 7.33-7.27 (m, 2H), 7.15-7.05 (m, 2H), 7.00-6.91 (m, 4H), 4.89-4.77 (m, 4H), 4.49-4.26 (m, 6H), 4.22-4.08 (m, 3H), 3.97-3.53 (m, 28H), 3.50-3.16 (m, 8H), 2.65 (s, 1H), 2.36-2.17 (m, 8H), 2.12-1.99 (m, 3H), 1.89-1.77 (m, 4H), 1.68-1.56 (m, 4H), 1.34-1.23 (m, 36H), 0.91-0.84 (m, 24H);

^{31}P NMR (162 MHz, chloroform-d): δ_P 59.25;

HRMS (ES+): calculated for $C_{88}H_{137}N_{27}O_6P_2$ 1731.0793 $[M+H]^+$, found 1731.0794 $[M+H]^+$.

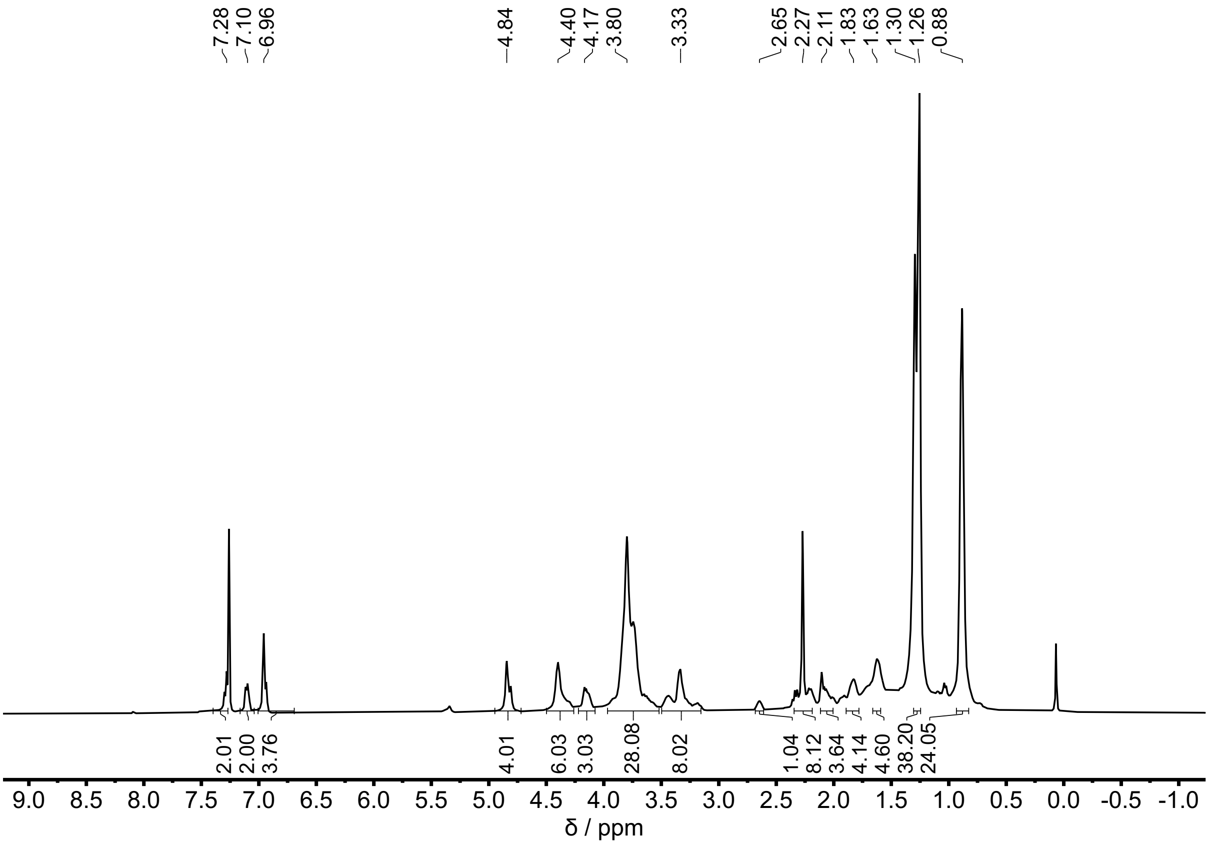

Figure S8: ^{1}H NMR (400 MHz, chloroform-d) spectrum of **zD*AD*Ay**.

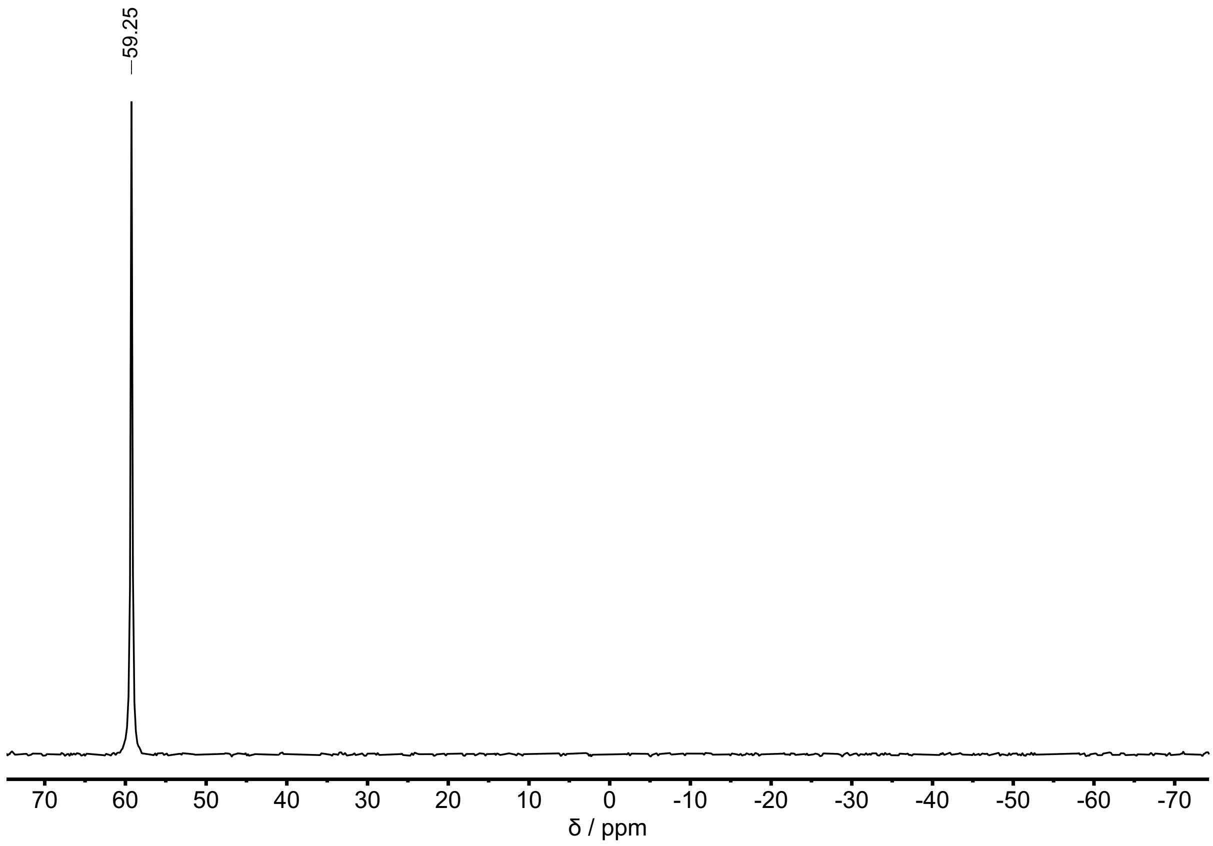

Figure S9: ^{31}P NMR (162 MHz, chloroform-d) spectrum of **zD*AD*Ay**.

Synthesis of pAp and pDp

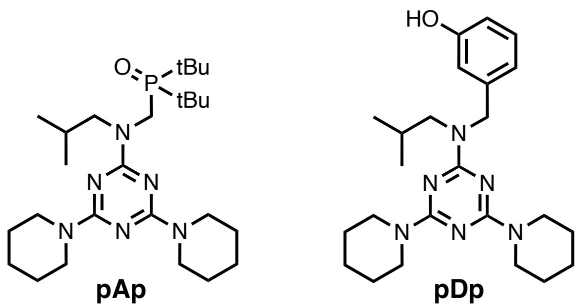

pAp and **pDp** were synthesised according to the literature procedures found at:

P. Troselj, P. Bolgar, P. Ballester, and C. A. Hunter, *J. Am. Chem. Soc.*, 2021, **143**, 8669–8678.

Synthesis of 8

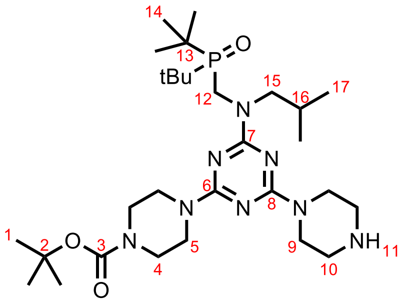

To a solution of **3** (826 mg, 2.09 mmol) in THF (9 mL) was added N-Boc-piperazine (389 mg, 2.09 mmol) and DIPEA (0.73 mL, 4.18 mmol) and the reaction was stirred at room temperature for 1 hour. Piperazine (1.8 g, 20.9 mmol) was added, and the reaction stirred in the microwave at 100 °C for 1 hour. The reaction was cooled to room temperature and diluted with EtOAc (30 mL) and washed with 1M sodium hydroxide (30 mL). The aqueous phase was extracted with EtOAc (3 x 30 mL) and the combined organic phases dried with magnesium sulphate. The solvent was evaporated *in vacuo*. The residues obtained were purified by flash chromatography (SiO_2, 0-20% gradient of methanol in DCM). **8** was obtained as a white foam (1.24 g, 2.09 mmol, quantitative).

^{1}H NMR (400 MHz, chloroform-d): δ_H 4.52 (br s, 1H, NH_{11}), 4.35 (br s, 2H, H_{12}), 3.90 (br s, 4H, H_9), 3.80 (d, J = 5.6 Hz, 2H, H_{15}), 3.71 (br, 4H, H_5), 3.43 (br, 4H, H_4), 3.01 (br, 4H, H_{10}), 2.17 (br, 1H, H_{16}), 1.47 (s, 9H, H_1), 1.26 (d, $^3J_{HP}$ = 12.9 Hz, 18H, H_{14}), 0.86 (d, J = 6.7 Hz, 6H, H_{17});

^{13}C NMR (101 MHz, chloroform-d): δ_C 165.5 and 165.5 ($C_{6,7,8}$), 154.9 (C_3), 80.2 and 80.1 (rotamers, C_2) 53.2 (C_{15}), 45.0 and 44.7 (rotamers, C_{10}), 43.1 ($C_{4,5}$), 42.6 and 42.2 (C_9), 38.1 (d, $^1J_{CP}$ = 59.7 Hz, C_{12}), 35.9 (d, $^1J_{CP}$ = 55.5 Hz, C_{13}), 28.6 (C_1), 26.9 (C_{14}), 26.5 (C_{16}), 20.7 (C_{17});

^{31}P NMR (162 MHz, chloroform-d): δ_P 58.88;

HRMS (ES+): Calculated for $C_{29}H_{56}N_8O_3P^+$, 595.4208; found 595.4210;

FT-IR (ATR): ν_{max}/cm^{-1} 3384 (broad), 2954, 2929, 2869, 1697, 1535, 1484, 1432, 1366, 1306, 1246, 1167, 1001, 834, 808.

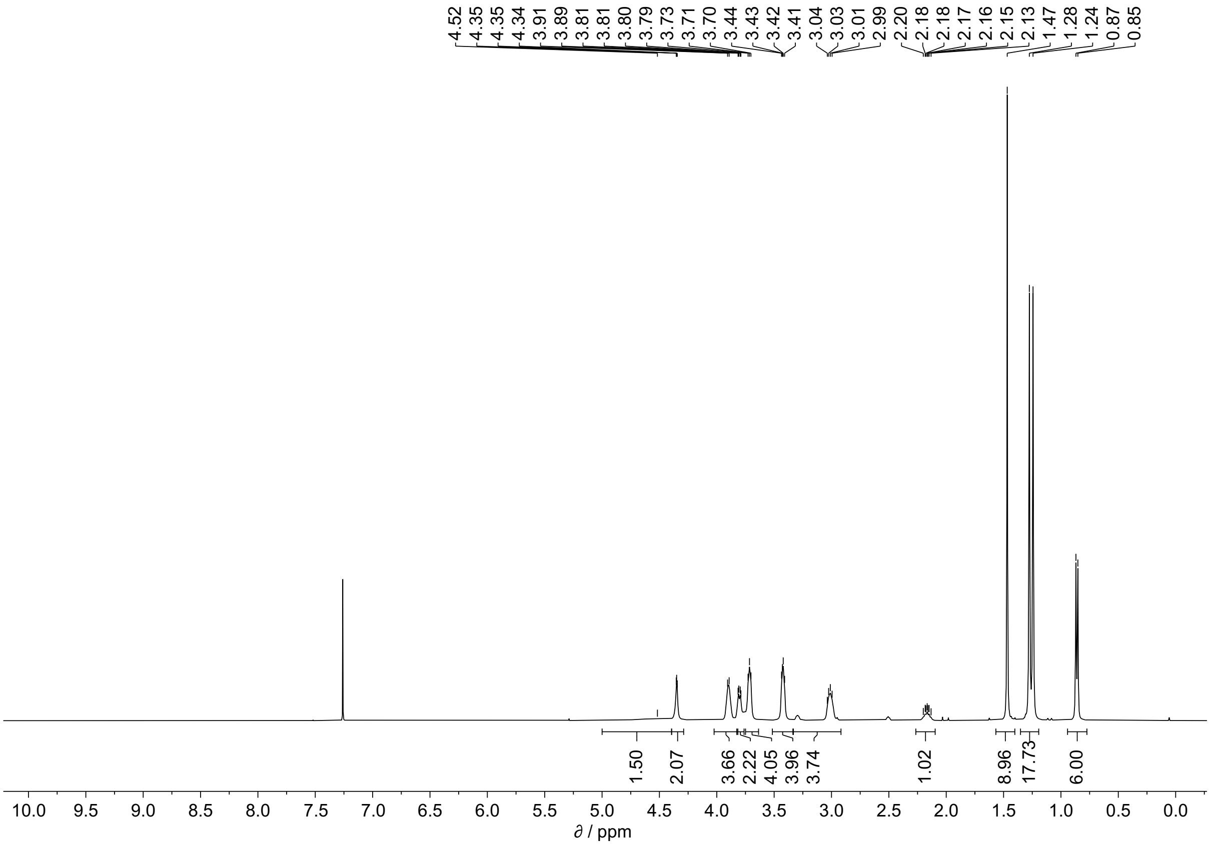

Figure S10: ^{1}H NMR (400 MHz, chloroform-d) spectrum of **8**.

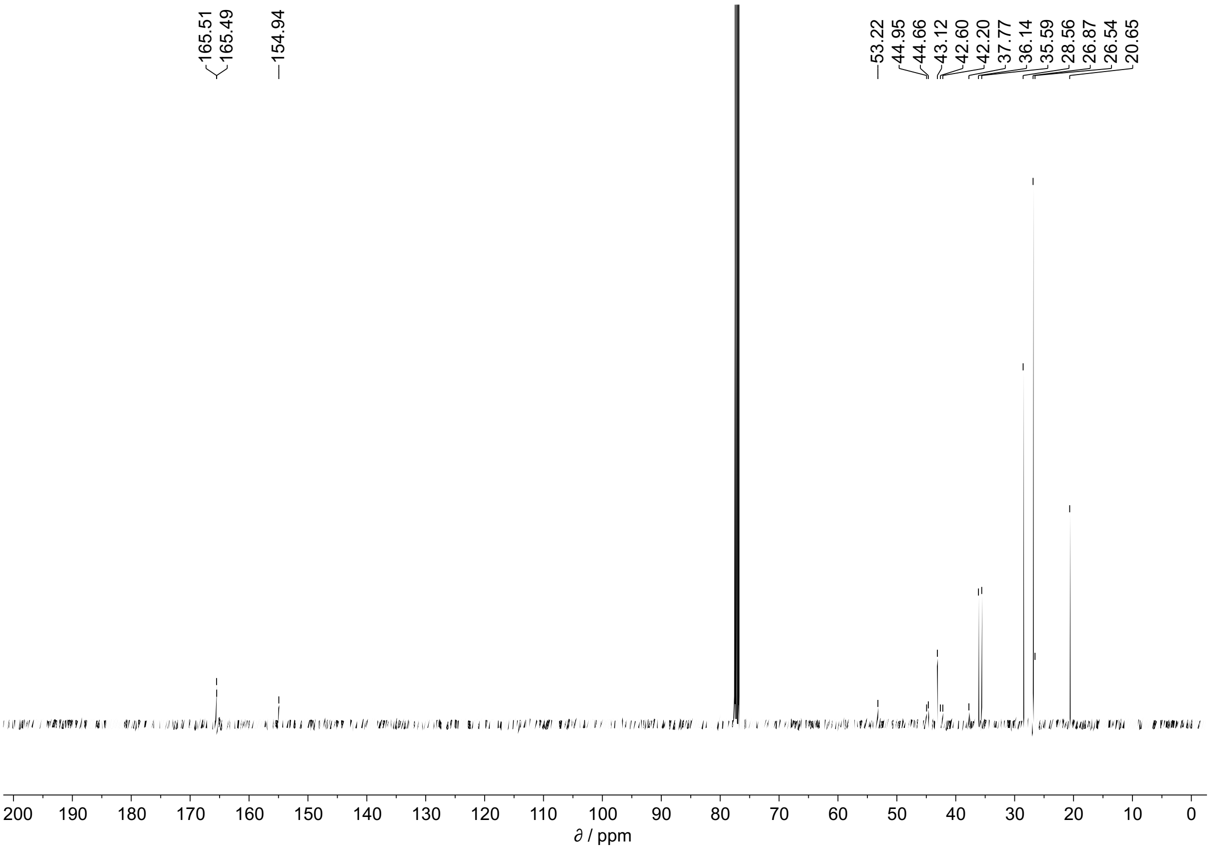

Figure S11: ^{13}C NMR spectrum (101 MHz, chloroform-d) of **8**.

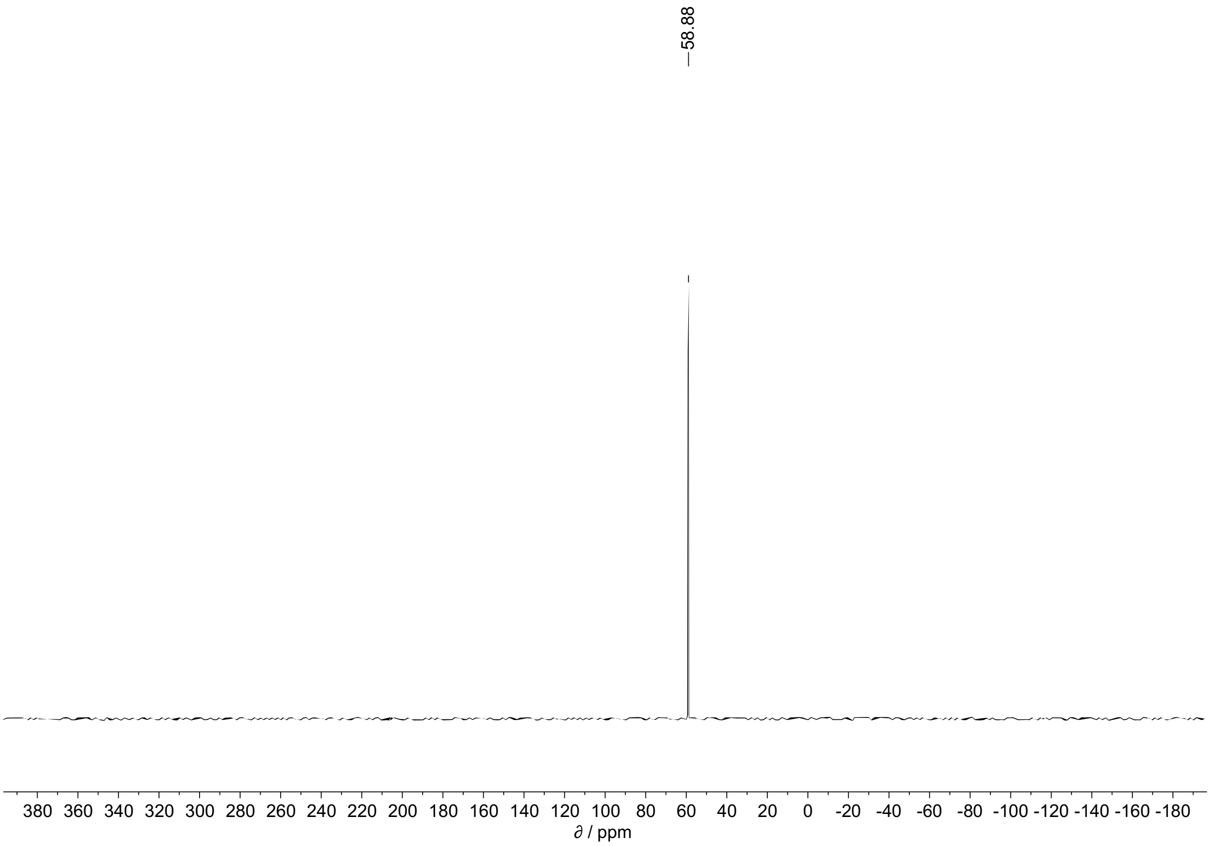

Figure S12: ^{31}P NMR spectrum (162 MHz, chloroform-d) of **8**.

Synthesis of n*AAAn*

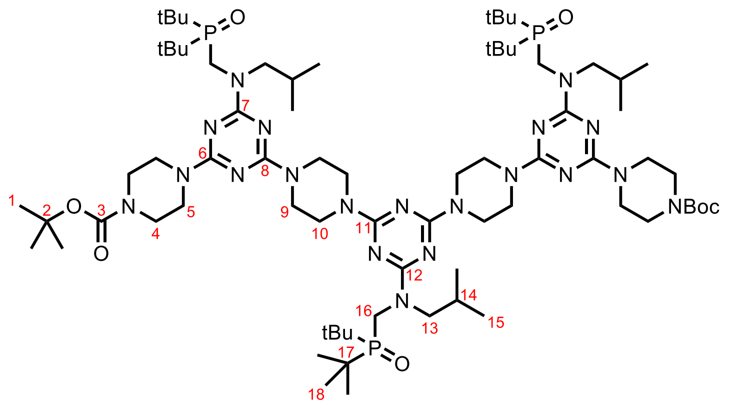

To a solution of **3** (375 mg, 0.948 mmol) in THF (4 mL), **8** (1.24 g, 2.08 mmol) and DIPEA (0.83 mL, 4.7 mmol) were added. The reaction was heated at 100 °C in the microwave for 1 hour. The reaction was cooled to room temperature and diluted with EtOAc (30 mL) and washed with 1M sodium hydroxide (30 mL). The aqueous phase was extracted with EtOAc (3 x 30 mL) and the combined organic phases dried with magnesium sulphate. The solvent was evaporated *in vacuo*. The residues obtained were purified by flash chromatography (SiO_2, 0-15% gradient of methanol in EtOAc). **n*AAAn*** was obtained as a white foam (991 mg, 0.655 mmol, 69%).

^{1}H NMR (400 MHz, chloroform-d): δ_H 4.37 (br, 6H, H_{16}), 3.84 (br, 6H, H_{13}), 3.77 (br s, 16H, $H_{9,10}$), 3.72 (br, 8H, H_5), 3.43 (br, 8H, H_4), 2.18 (m, 3H, H_{14}), 1.46 (s, 18H, H_1), 1.26 (2 doublets, $^3J_{HP}$ = 13.0 Hz, 54H, H_{18}), 0.87 (2 doublets, J = 6.7 Hz, 18H, H_{15});
^{13}C NMR (101 MHz, chloroform-d): δ_C 165.5 ($C_{6,7,8,11,12}$), 154.9 (C_3), 80.1 (C_2), 53.1 (C_{13}), 43.14 ($C_{4,5,9,10}$), 38.1 (d, $^1J_{CP}$ = 58.9 Hz, C_{16}), 35.8 (d, $^1J_{CP}$ = 55.5 Hz, C_{17}), 28.5 (C_1), 26.9 (C_{18}), 26.5 (C_{14}), 20.6 (C_{15});
^{31}P NMR (162 MHz, chloroform-d): δ_P 58.67;
HRMS (ESI+): Calculated for $C_{74}H_{138}N_{20}O_7P_3^+$, 1512.0265; found 1512.0238;
FT-IR (ATR): ν_{max}/cm^{-1} 2954, 2928, 2903, 2868, 1696, 1525, 1476, 1427, 1364, 1246, 1160, 1080, 996, 832, 806, 731, 646, 581, 504, 449.

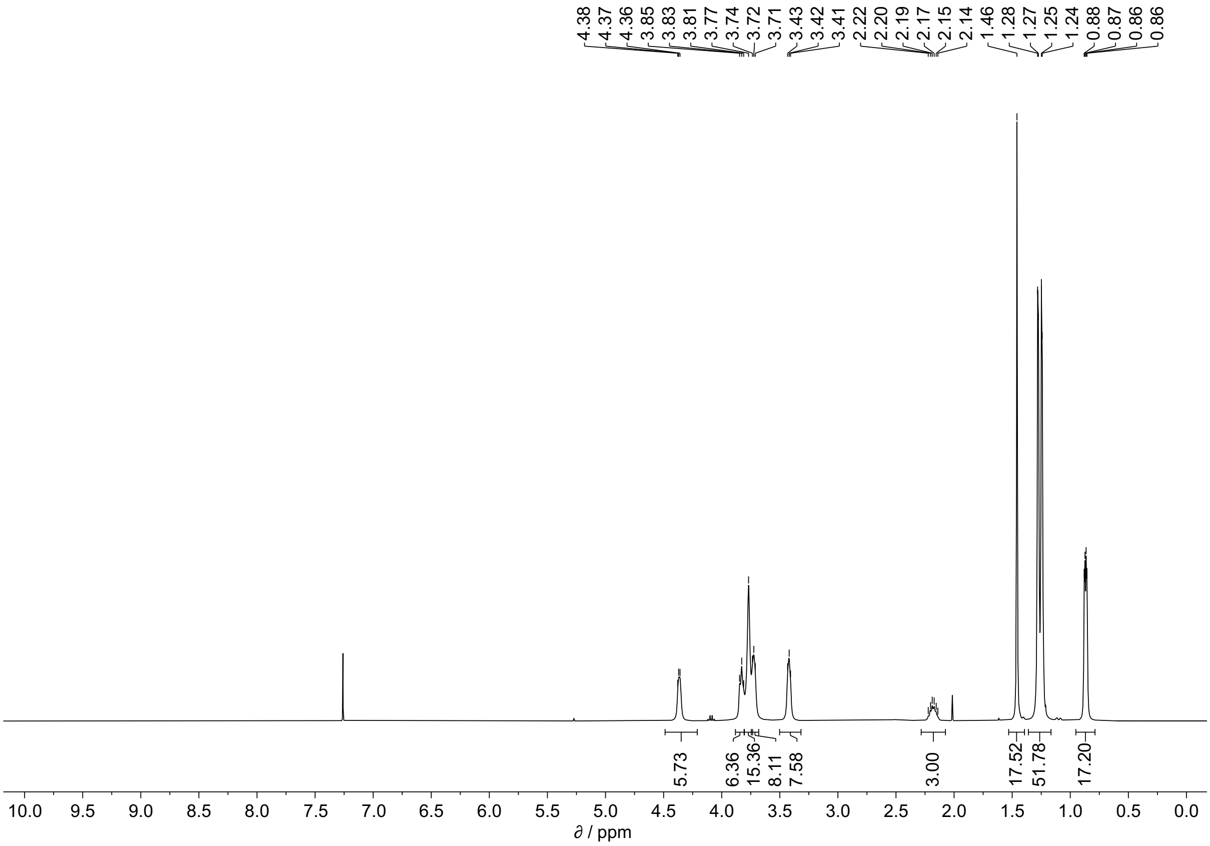

Figure S13: ^{1}H NMR spectrum (400 MHz, chloroform-d) of **n*AAAn***.

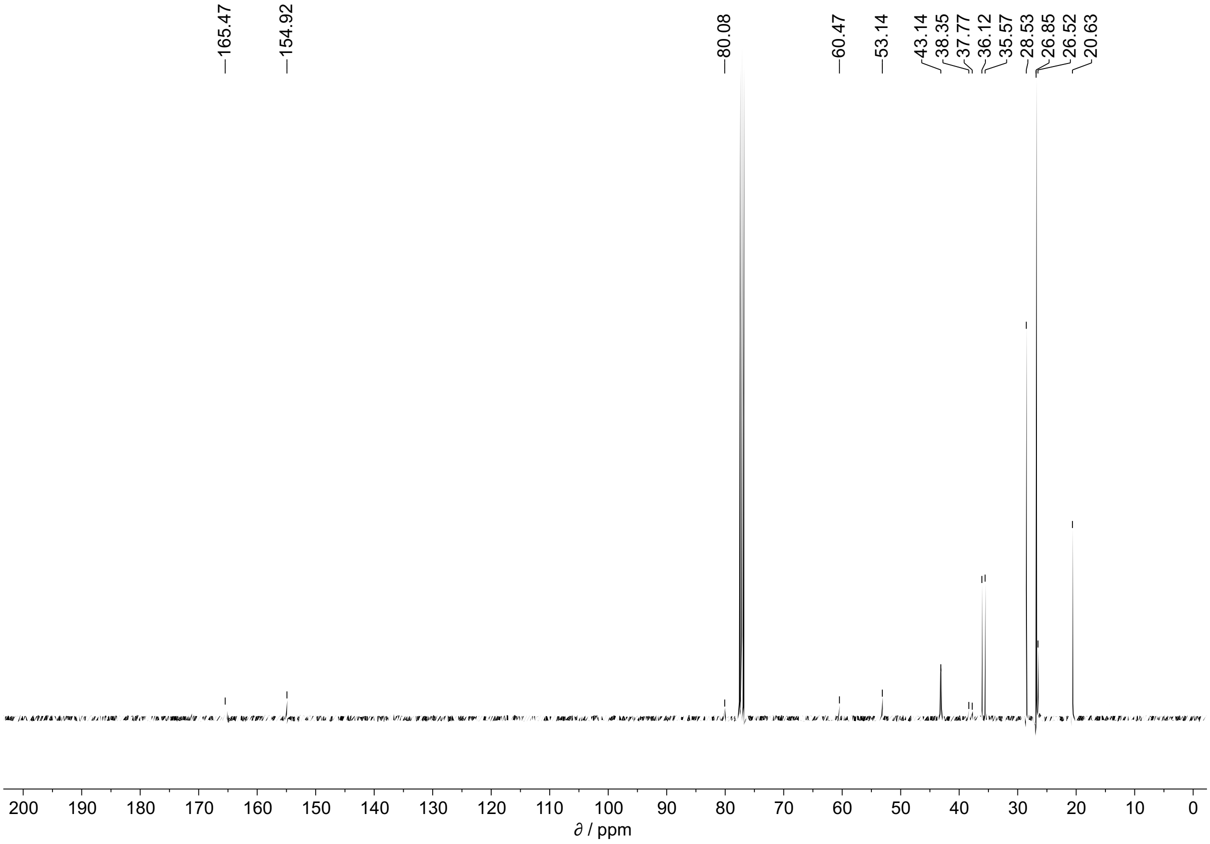

Figure S14: ^{13}C NMR spectrum (101 MHz, chloroform-d) of **n*AAAn***.

.

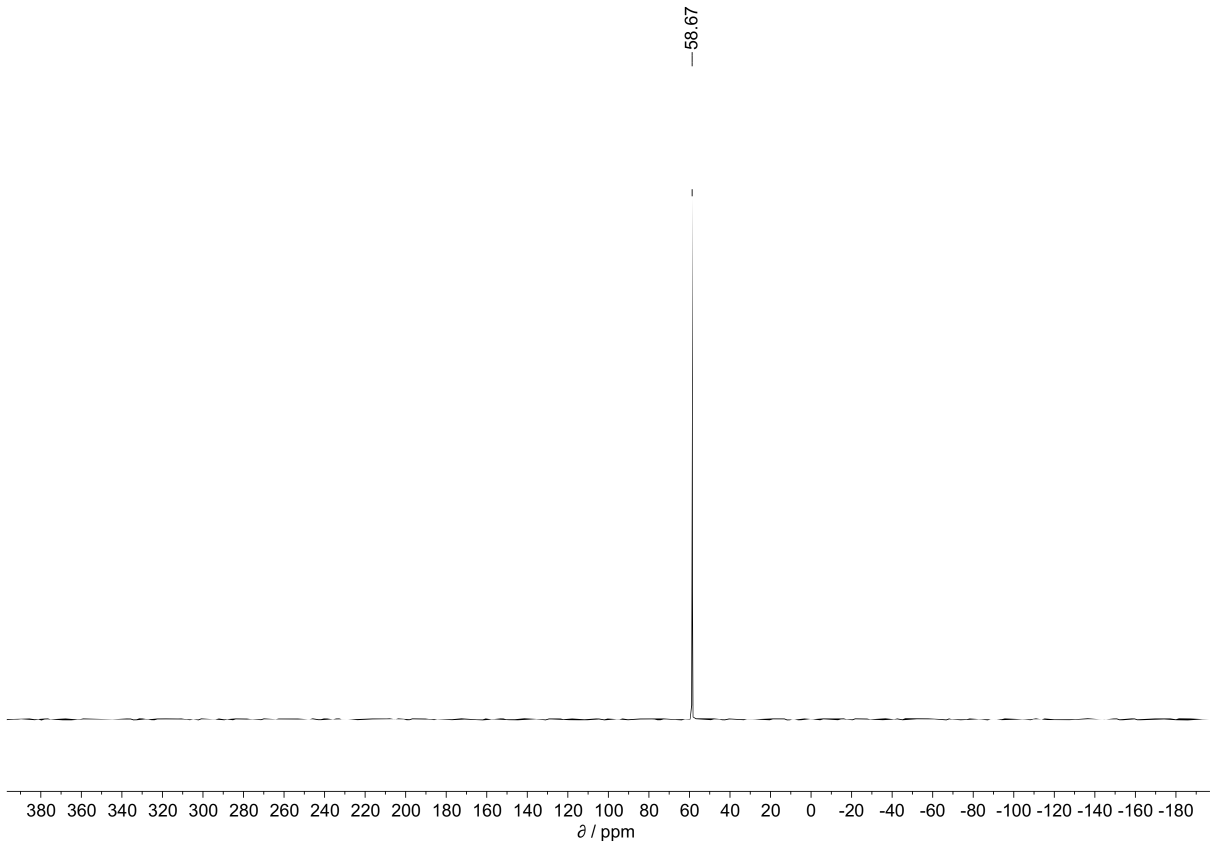

Figure S15: ^{31}P NMR spectrum (162 MHz, chloroform-d) of **n*AAAn***.

Synthesis of 9

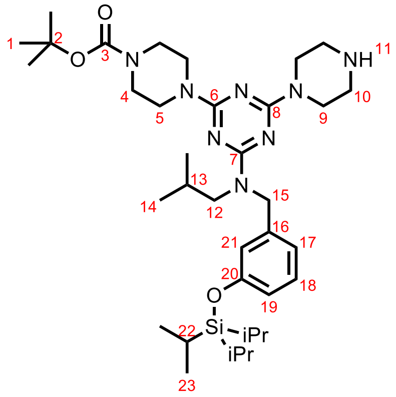

To a solution of **1** (2.04 g, 4.2 mmol) in THF (18 mL) was added N-Boc-piperazine (782 mg, 4.2 mmol) and DIPEA (1.46 mL, 8.4 mmol) and the reaction was stirred at room temperature for 1 hour. Piperazine (7.2 g, 84 mmol) was added, and the reaction stirred in the microwave at 100 °C for 1 hour. The reaction was cooled to room temperature and diluted with EtOAc (50 mL) and washed with 1M sodium hydroxide (50 mL). The aqueous phase was extracted with EtOAc (3 x 30 mL) and the combined organic phases dried with magnesium sulphate. The solvent was evaporated *in vacuo***.** The residues obtained were purified by flash chromatography (SiO_2, 0-10% gradient of methanol in DCM). **9** was obtained as a white foam (2.44 g, 3.6 mmol, 85%).

^{1}H NMR (400 MHz, chloroform-d): δ_H 7.11 (t, J = 7.8 Hz, 1H, H_{18}), 6.78 (d, J = 7.5 Hz, 1H, H_{17}), 6.73 (dd, J = 7.9, 1.7 Hz, 1H, H_{19}), 6.69 (s, 1H, H_{21}), 4.75 (s, 2H, H_{15}), 3.72 (br, 8H, $H_{5,9}$), 3.44 and 3.37 (t, J = 4.9 Hz, 4H, rotamers, H_4), 3.27 (d, J = 7.2 Hz, 2H, H_{12}), 2.91 and 2.84 (t, J = 4.8 Hz, 4H, rotamers, H_{10}), 2.52 (br s, 1H, H_{11}), 2.06 (non, J = 6.7 Hz, 1H, H_{13}), 1.48 and 1.46 (s, 9H, rotamers, H_1), 1.16 (m, 3H, H_{22}), 1.04 (d, J = 7.2 Hz, 18H, H_{23}), 0.87 (d, J = 6.7 Hz, 6H, H_{14});

^{13}C NMR (101 MHz, chloroform-d): δ_C 166.3 (C_7), 165.5 ($C_{6/8}$) 165.4 ($C_{6/8}$), 156.3 (C_{20}), 155.0 and 155.0 (rotamers, C_3), 140.9 (C_{16}), 129.2 (C_{18}), 120.2 (C_{17}), 118.7 and 118.6 (rotamers, C_{21}), 118.4 (C_{19}), 79.9 and 79.9 (rotamers, C_2), 53.7 and 53.7 (rotamers, C_{12}), 50.2 and 50.2 (rotamers, C_{15}), 45.9 and 45.8 (rotamers, C_{10}) 44.0 and 43.9 (rotamers, $C_{5,9}$), 43.1 and 43.1 (rotamers, C_4), 28.6 (C_1), 27.6 (C_{13}), 20.7 (C_{14}), 18.0 (C_{23}), 12.8 (C_{22});

HRMS (ESI+): Calculated for $C_{36}H_{63}N_8O_3Si^+$, 683.4787; found 683.4784;

FT-IR (ATR): ν_{max}/cm^{-1} 2944, 2865, 1698, 1602, 1586, 1531, 1481, 1439, 1418, 1388, 1365, 1341, 1310, 1275, 1245, 1233, 1205, 1167, 1107, 1074, 1001, 883, 867, 827, 808, 778, 688.

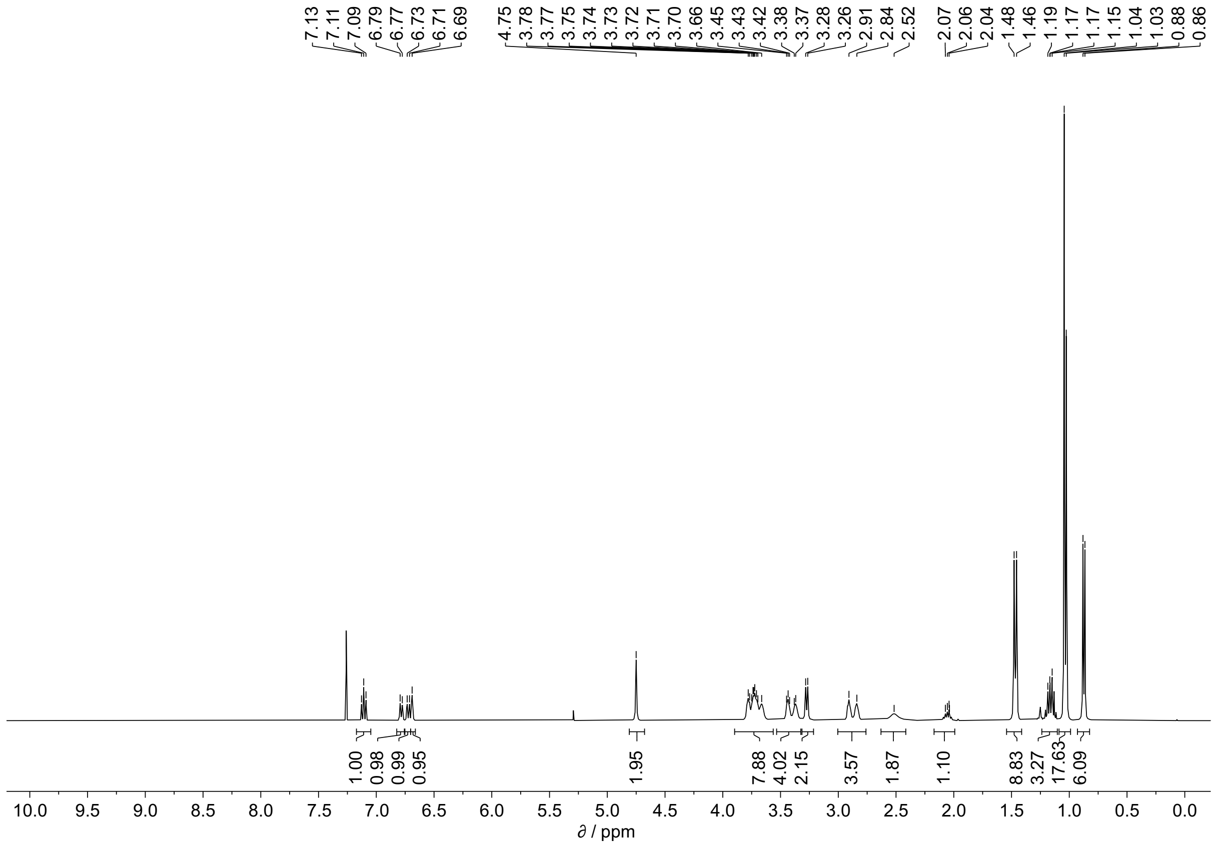

Figure S16: ^{1}H NMR spectrum (400 MHz, chloroform-d) of **9**.

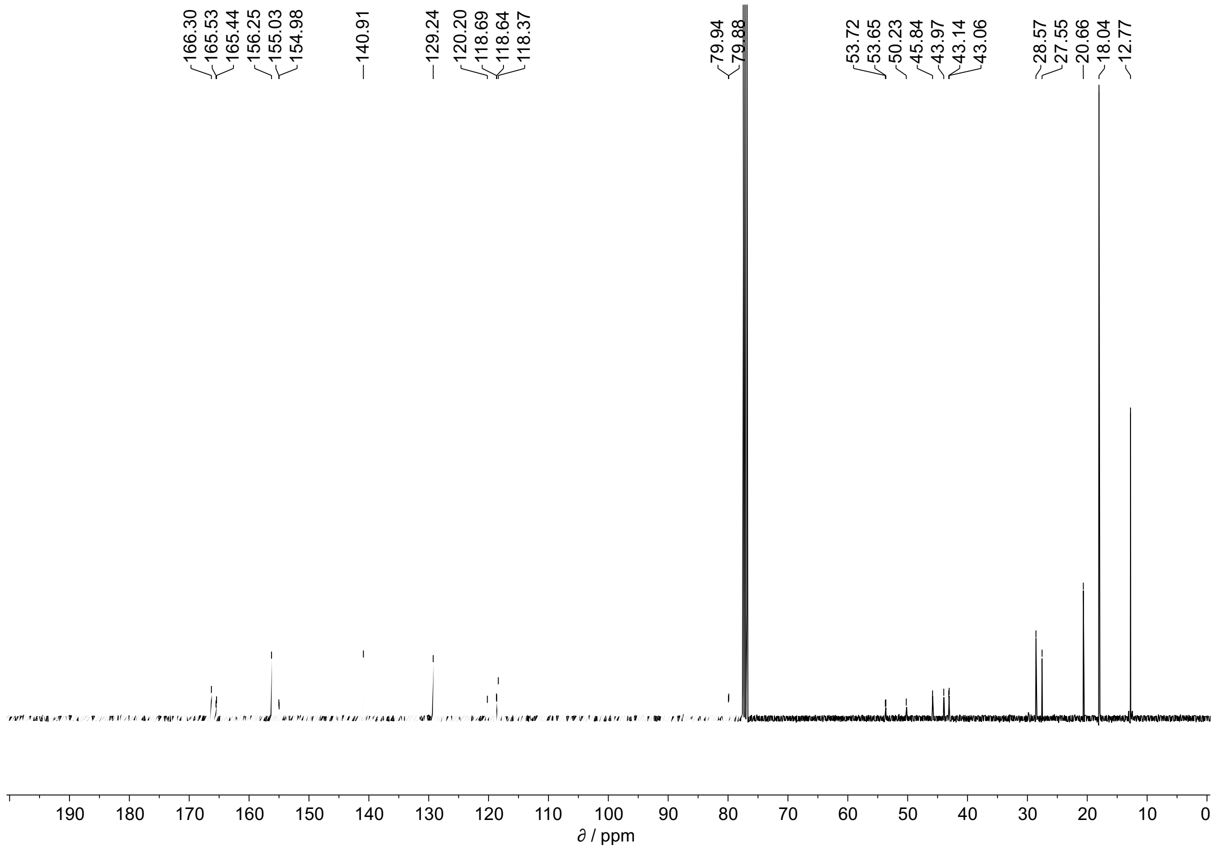

Figure S17: ^{13}C NMR spectrum (101 MHz, chloroform-d) of **9**.

Synthesis of 10

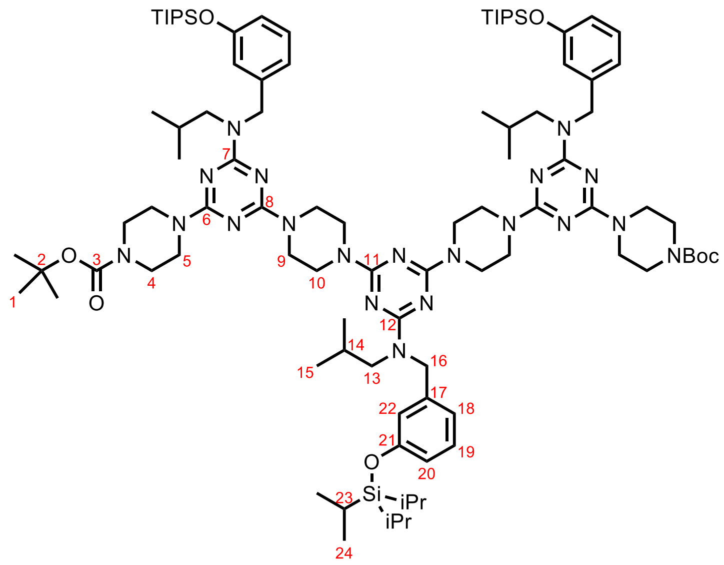

To a solution of **1** (569 mg, 1.18 mmol) in THF (7 mL), **9** (2.42 mg, 3.55 mmol) and DIPEA (3.1 mL, 17.8 mmol) were added. The reaction was heated at 100 °C in the microwave for 1 hour. The reaction was cooled to room temperature and diluted with EtOAc (30 mL) and washed with 1M sodium hydroxide (30 mL). The aqueous phase was extracted with EtOAc (3 x 30 mL) and the combined organic phases dried with magnesium sulphate. The solvent was evaporated *in vacuo.* The residues obtained were purified by flash chromatography (SiO_2, 0-10% gradient of DCM in EtOAc, then 0-10% gradient of methanol in DCM). **10** was obtained as a white foam (1.91 g, 1.07 mmol, 91%).

^{1}H NMR (400 MHz, chloroform-d): δ_H 7.11 (br, 3H, H_{19}), 6.80 (br, 3H, H_{18}), 6.72 (m, 6H, $H_{20,22}$), 4.78 (br, 6H, H_{16}), 3.87 – 3.51 (br, 24H, $H_{4/5/9/10}$), 3.45 and 3.38 (br s, 8H, rotamers, $H_{4/5/9/10}$), 3.30 (br, 6H, H_{13}), 2.07 (br, 3H, H_{14}), 1.49 and 1.47 (s, 18H, rotamers, H_1), 1.16 (m, 9H, H_{23}), 1.03 (m, 54H, H_{24}), 0.89 (m, 18H, H_{15});
^{13}C NMR (101 MHz, chloroform-d): δ_C 166.2 (C_{12}), 165.4 (C_{11}), 156.1 (C_{21}), 154.9 and 154.8 (rotamers, C_{17}), 140.8 (C_{17}), 129.1 (C_{19}), 120.0 (C_{18}), 118.5 ($C_{20/22}$), 118.2 ($C_{20/22}$), 79.8 and 79.7 (rotamers, C_2), 53.5 ($C_{4/5/9/10}$), 50.0 (C_{16}), 43.0 ($C_{13,4/5/9/10}$), 28.4 (C_1), 27.4 (C_{14}), 20.5 (C_{15}), 17.9 (C_{24}), 12.6 (C_{23});
HRMS (ESI+): Calculated for $C_{95}H_{159}N_{20}O_7Si_3^+$, 1776.2003; found 1776.1953;
FT-IR (ATR): ν_{max}/cm^{-1} 2944, 2865, 1699, 1602, 1587, 1528, 1479, 1419, 1365, 1245, 1204, 1168, 996, 883, 807, 778, 738, 687.

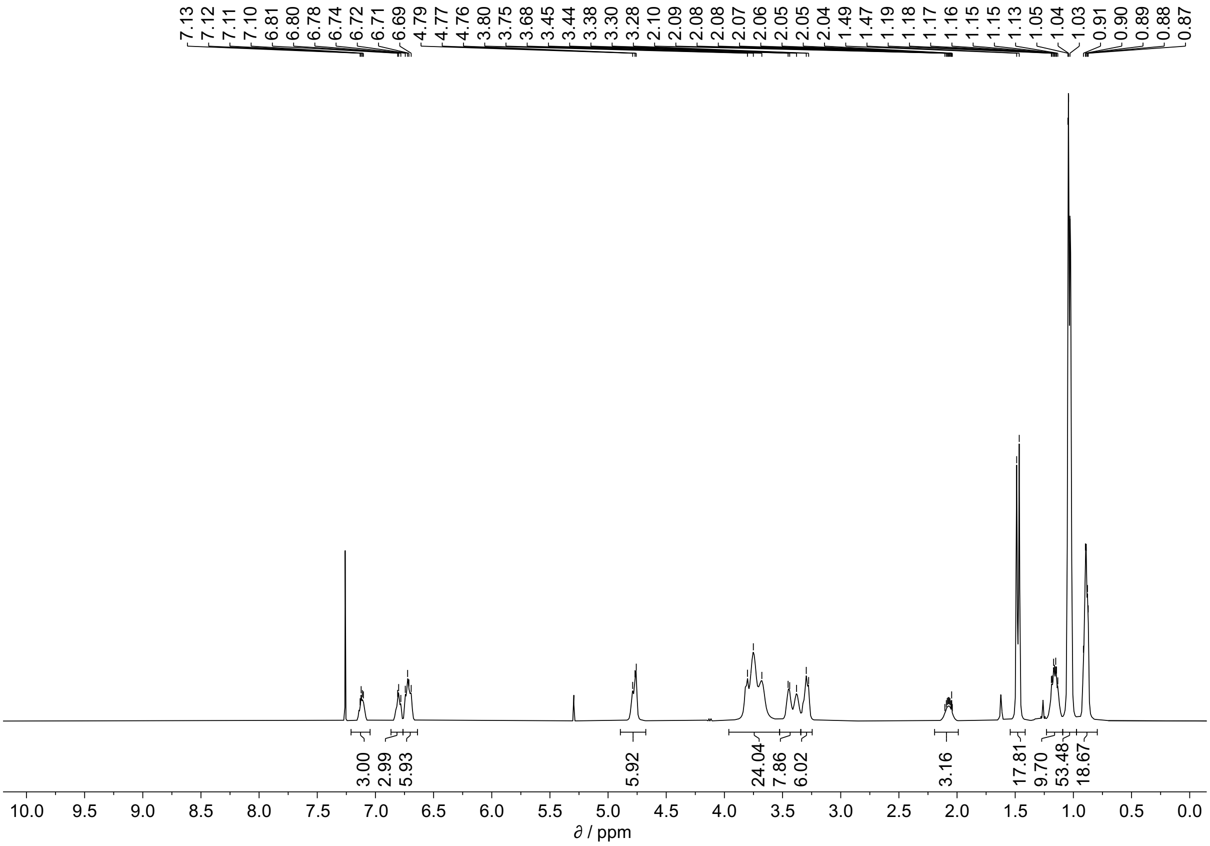

Figure S18: ^{1}H NMR spectrum (400 MHz, chloroform-d) of **10**.

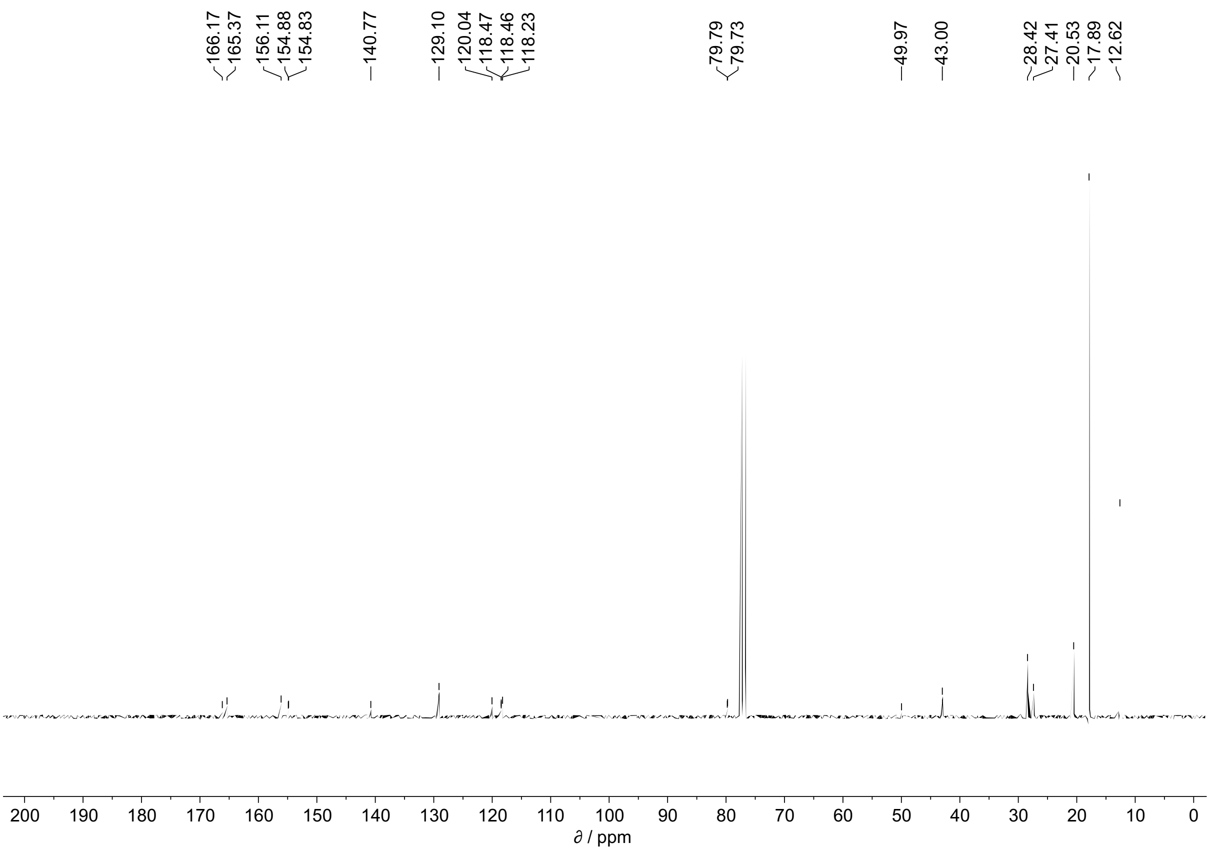

Figure S19: ^{13}C NMR spectrum (101 MHz, chloroform-d) of **10**.

Synthesis of n*DDDn*

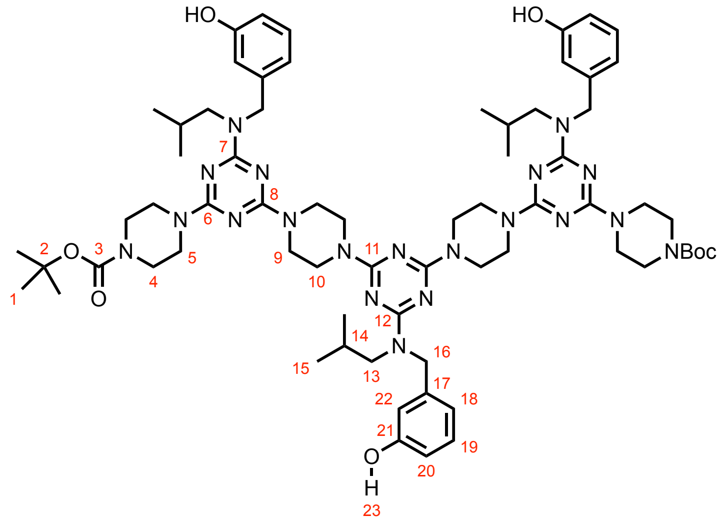

To a solution of **10** (110 mg, 0.062 mmol) in dry THF (20 mL) at room temperature, TBAF solution (1M in THF) was added dropwise in 3 portions, with stirring and a 5 minute interval after each addition. 5 minutes after the final addition, the reaction was quenched with water (30 mL) and diluted with EtOAc (50 mL). The organic phase was collected. The aqueous phase was washed with EtOAc (3 x 30 mL) and the combined organic phases dried with magnesium sulphate. The solvent was evaporated *in vacuo*. The residues obtained were purified by flash chromatography (SiO_2, 0-10% gradient of methanol in DCM). **n*DDDn*** was obtained as a white foam (71 mg, 0.054 mmol, 89%).

^{1}H NMR (700 MHz, chloroform-d): δ_H 7.11 (br, 3H, H_{19}), 6.75 (br, 3H, H_{18}), 6.66 (m, 6H, $H_{20,22}$), 6.02 (br s, 3H, H_{23}), 4.76 (br, 6H, H_{16}), 3.82-3.64 (br, 24H, $H_{4/5/9/10}$), 3.43 and 3.37 (br s, 8H, rotamers, $H_{4/5/9/10}$), 3.31 (br, 6H, H_{13}), 2.07 (br, 3H, H_{14}), 1.48 and 1.46 (s, 18H, rotamers, H_1), 0.88 (m, 18H, H_{15});
^{13}C NMR (176 MHz, chloroform-d): δ_C 166.2 (C_{12}), 165.5 (C_{11}), 156.2 (C_{21}), 155.2 (C_{17}), 141.4 (C_{17}), 129.5 (C_{19}), 119.6 (C_{18}), 114.4 ($C_{20/22}$), 113.8 ($C_{20/22}$), 80.3 (C_2), 53.7 ($C_{4/5/9/10}$), 50.2 (C_{16}), 43.2 and 43.1 (rotamers, $C_{13,4/5/9/10}$), 28.6 (C_1), 27.5 (C_{14}), 20.7 (C_{15});
HRMS (ESI+): Calculated for $C_{68}H_{98}N_{20}O_7$, 1307.8005; found 1307.8018;
FT-IR (ATR): ν_{max}/cm^{-1} 2919, 2865, 1699, 1670, 1591, 1533, 1484, 1426, 1387, 1367, 1259, 1206, 1170, 998, 806.

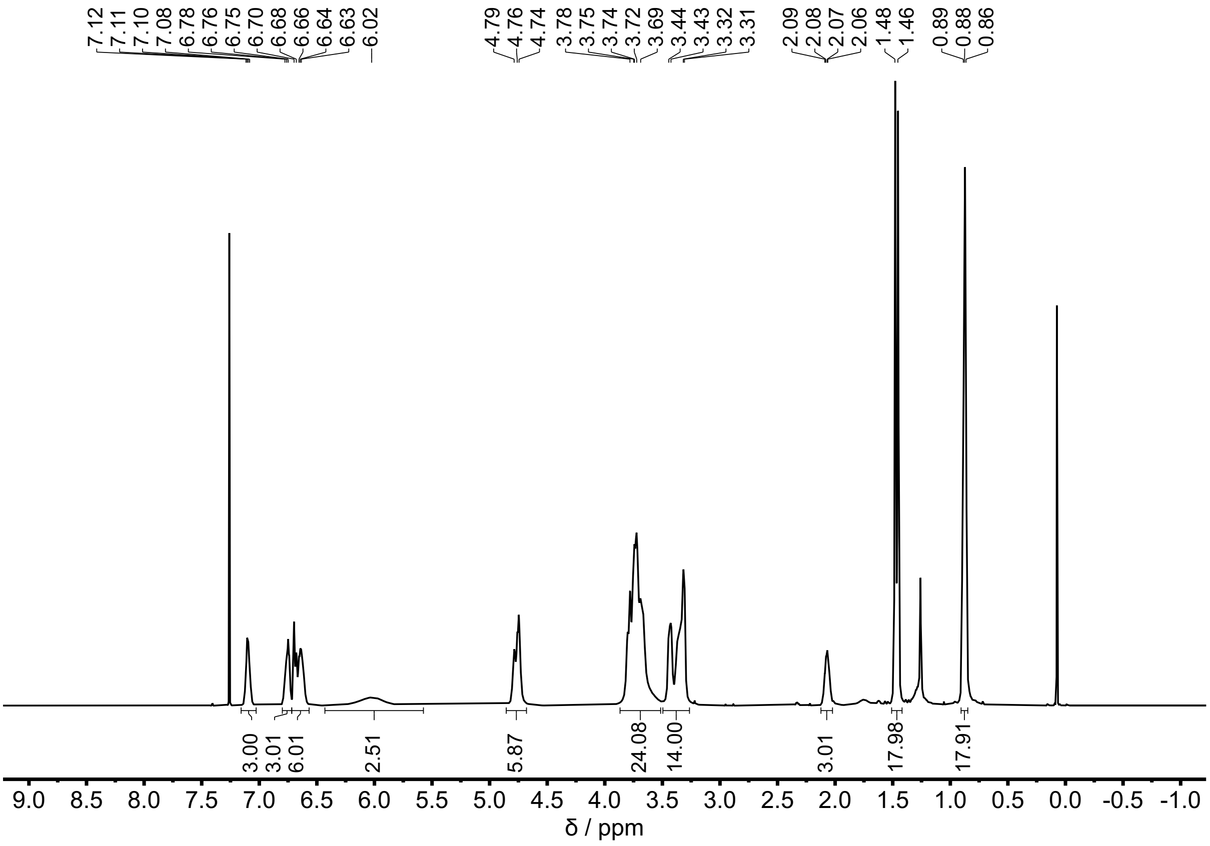

Figure S20: ^{1}H NMR spectrum (700 MHz, chloroform-d) of **n*DDDn***.

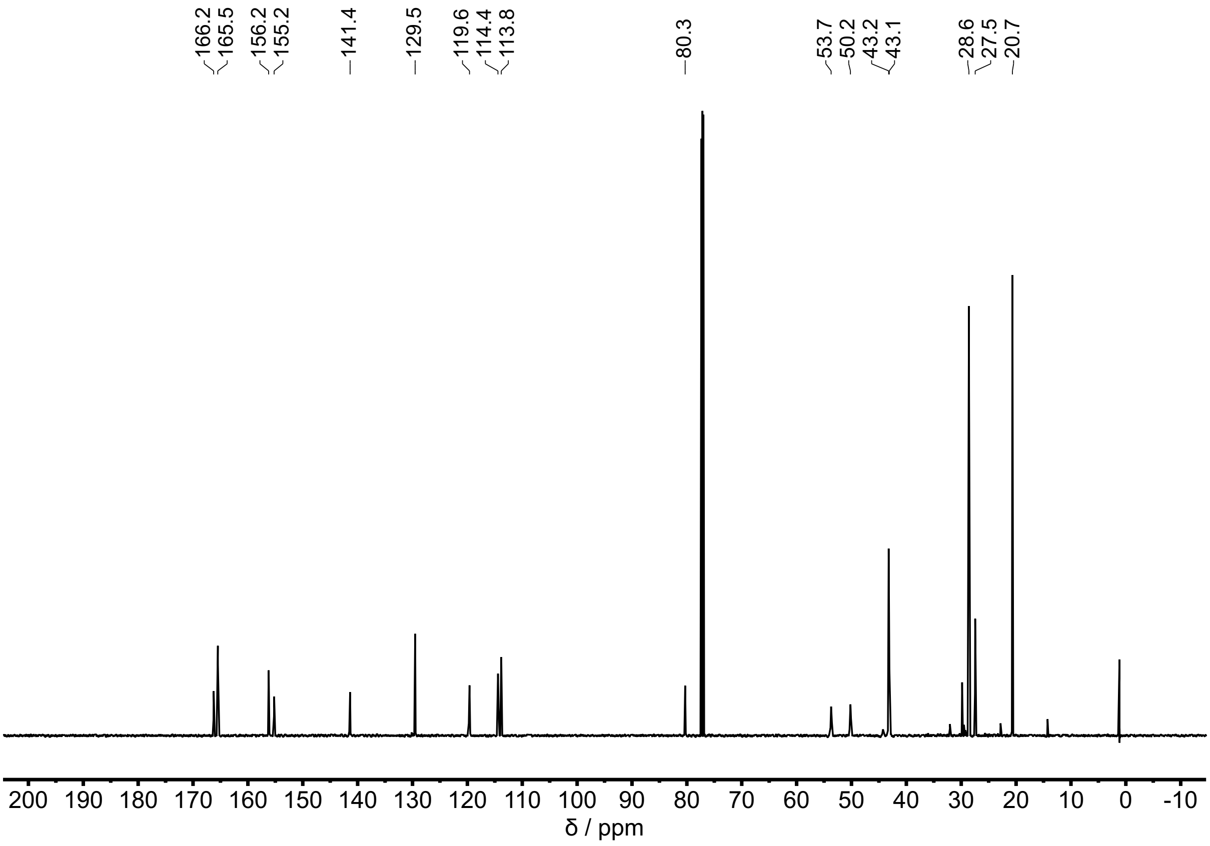

Figure S21: ^{13}C NMR spectrum (176 MHz, chloroform-d) of **n*DDDn***.

6. ITC Dilution of pDADAp

To study dimerisation, **pDADAp** was dissolved in HPLC grade chloroform with a concentration approximately 10 times the expected dissociation constant and loaded into the injection syringe. Pure solvent was loaded into the sample cell of the microcalorimeter. The number of injections was 35, and the volume of the injections was 8 μL. The thermogram peaks were integrated using Microcal Origin, and the resulting data were fit to a dimerization isotherm using purpose-written macros in Microsoft Excel. The macros use an iterative procedure to fit the experimental data to the following equations to determine the optimum values for the association constant (*K*) and the enthalpy of dimerization (Δ*H*°). Errors are quoted as two standard deviations based on three different experiments.

$$[\mathrm{Gt}]_s = [\mathrm{G}]_s + 2[\mathrm{G} \bullet \mathrm{G}]_s \quad (1)$$

$$[\mathrm{G} \bullet \mathrm{G}]_s = K[\mathrm{G}]_s^2 \quad (2)$$

where $[\mathrm{Gt}]_s$ is the total concentration in the syringe,

$[\mathrm{G}]_s$ is the concentration of monomeric species in the syringe,

$[\mathrm{G} \bullet \mathrm{G}]_s$ is the concentration of dimer in the syringe.

$$[\mathrm{Gt}]_i = [\mathrm{G}]_i + 2[\mathrm{G} \bullet \mathrm{G}]_i \quad (3)$$

$$[\mathrm{G} \bullet \mathrm{G}]_s = K[\mathrm{G}]_i^2 \quad (4)$$

where $[\mathrm{Gt}]_i$ is the total concentration in the cell after the ith injection,

$[\mathrm{G}]_i$ is the concentration of monomeric species in the cell after the ith injection,

$[\mathrm{G} \bullet \mathrm{G}]_i$ is the concentration of dimer in the cell after the ith injection.

The integrated molar heat of the ith injection (Q_i) is given by:

$$Q_i = Q_0 + 2\frac{V([\mathrm{G} \bullet \mathrm{G}]_i - [\mathrm{G} \bullet \mathrm{G}]_{i-1}) + V_i([\mathrm{G} \bullet \mathrm{G}]_{i-1} - [\mathrm{G} \bullet \mathrm{G}]_s)}{V_i[Gt]_s}\Delta H^\circ \quad (5)$$

where Q_0 is the baseline correction that is usually of the order 1 kJ mol^{-1},

V is the volume of the cell, V_i is the volume of the ith injection.

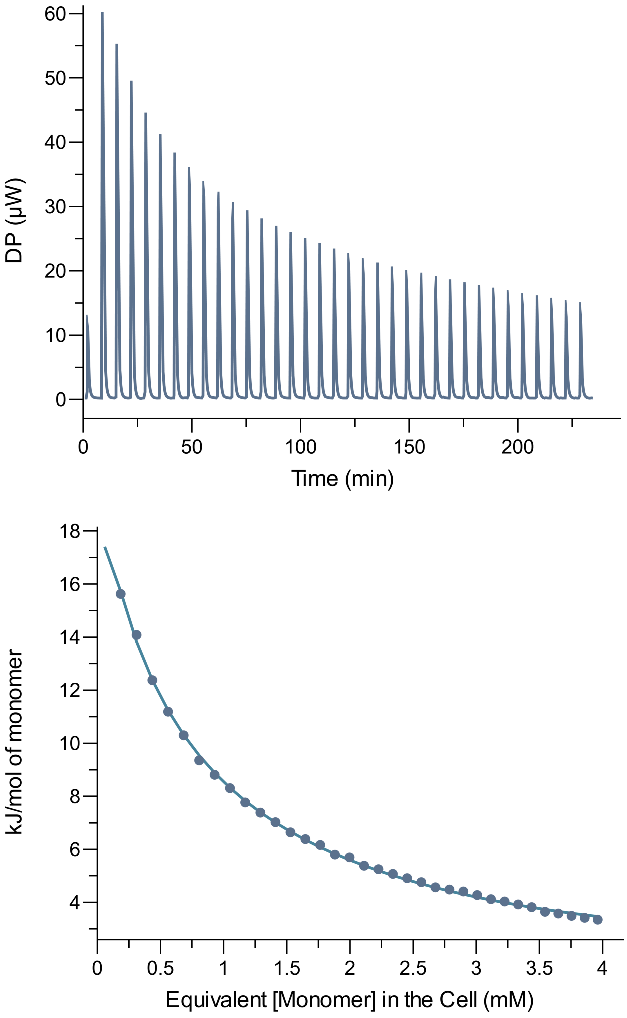

Figure S22: (a) ITC raw heat data (differential power, DP) for the dilution of **pDADAp** (22.9 mM in syringe) into chloroform at 298 K. **(b)** Fitted ITC data for the dilution of **pDADAp** (22.9 mM in syringe) into chloroform at 298K. Determined $K = 300 \pm 50$ M^{-1}, $\Delta H° = -43 \pm 2$ kJ mol^{-1}.

7. ^{31}P NMR Experiments

NMR Titrations

Homo-oligomer binding constants were measured by ^{31}P NMR titrations in a Bruker 400 MHz AVII HD Smart Probe spectrometer. The host (**n*AAAn***) was dissolved in $CDCl_3$ at a known concentration. The guest (**n*DDDn***) was dissolved in the host solution and made to a known concentration. 0.6 mL of host was added to an NMR tube and the spectrum was recorded. Aliquots of guest in host solution were added to the NMR tube, and the spectra were recorded after each addition. The chemical shifts of the host spectra were monitored as a function of guest concentration and analysed using *Musketeer* (available from the GitHub repository, https://github.com/daniilS/Musketeer/releases). The changes in chemical shift were fit to a 1:1 binding isotherm. Errors are quoted as two standard deviations based on three different experiments.

NMR Titration of n*DDDn* into n*AAAn*

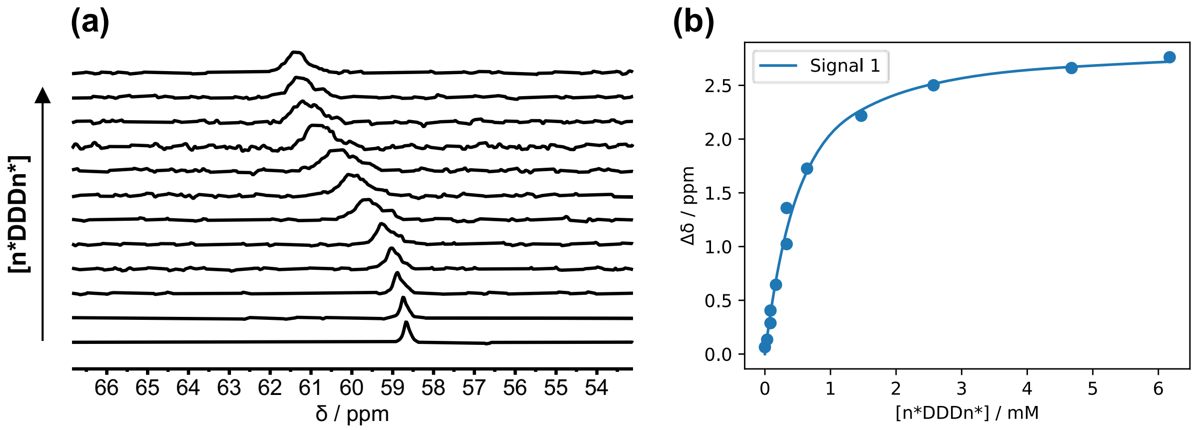

Figure S23: (a) ^{31}P NMR spectra (162 MHz) for the titration of **n*DDDn*** into **n*AAAn*** (0.96 mM in $CDCl_3$, 298K). **(b)** Plot of the change in chemical shift of the ^{31}P NMR signal as a function of guest concentration, where the line represents the best fit to a 1:1 binding isotherm allowing for variable guest concentration with $K = 3100 \pm 100$ M^{-1}, $\delta_{free} = 58.6$ ppm and $\delta_{bound} = 61.3$ ppm.

Thermal Denaturation of zDADAy•zDADAy Complex

Thermal denaturation data was measured by making a solution of **zDADAy** at 1 mM concentration in 1,1,2,2-tetrachloroethane-d_2 and measuring ^{31}P NMR spectra at different temperatures in a Bruker 400 MHz Neo Prodigy spectrometer equipped with a BCU Chiller unit. The temperature of the sample was changed using the internal thermostat of the NMR spectrometer, and the sample was allowed to equilibrate in the probe until the probe thermometer gave a stable temperature. The ^{31}P NMR chemical shifts of the acceptor homo-oligomer spectra were monitored as a function of temperature. Due to significant broadening of the ^{31}P NMR peaks, 40 Hz line broadening was applied to all spectra.

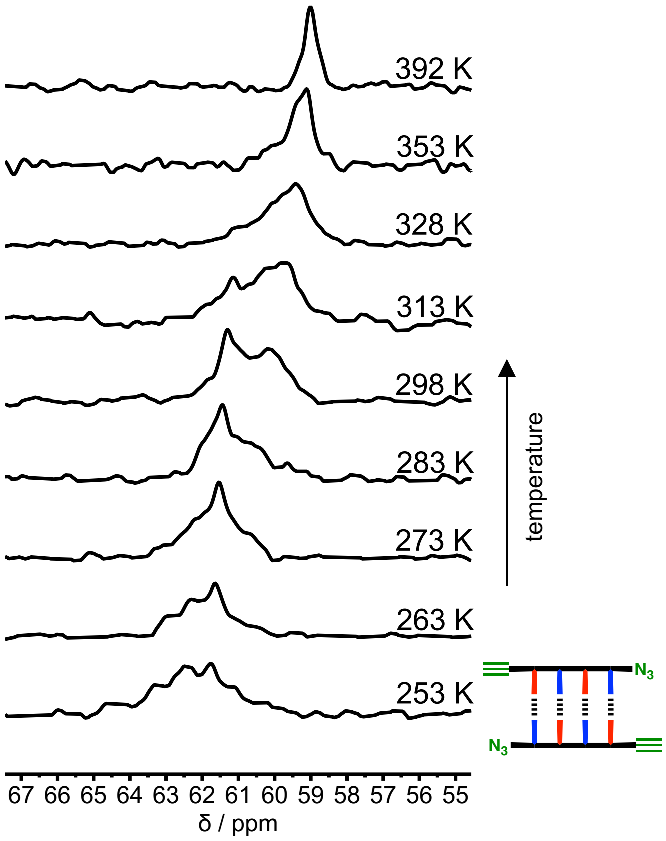

Figure S24: Variable temperature ^{31}P NMR spectra (162 MHz) for a 1 mM solution of **zDADAy** in TCE-d_2. Line broadening of 40 Hz was applied to all spectra.

8. CuAAC Duplex Trapping Experiments

General Procedure for CuAAC Trapping Experiments

For each experiment, fresh stock solutions of oligomers, 4-*t*-butylbenzyl azide and $Cu(MeCN)_4PF_6$-TBTA in dry DCM were prepared. The calculated amount from stock solutions of oligomers and 4-*t*-butylbenzyl azide were transferred to a reaction vial and the solvent was removed via evaporation under a stream of N_2. Cu(I)-TBTA in dry DCM was then added to the reaction vial under a stream of N_2, and mixtures were left to stir for 48 h at r.t. After reaction completion, the DCM was evaporated under a stream of N_2 and mixtures were re-dissolved in THF/MeOH to a known concentration, then sonicated to ensure that the products were fully dissolved. Mixtures were injected into the LCMS (between 2-5 µL injections) and UV peaks were identified (λ 254 nm). Peak identity was assigned based on the corresponding mass spectra and retention time. Two different UPLC gradients were used for analysis of product mixtures.

UPLC Peak Areas

Figure reference	Normalised peak area			
	macrocyclic single strand	linear single strand	macrocyclic duplex	linear duplex
Fig. 6b	0.18	0.82	-	-
Fig. 6c	0.09	0.28	0.37	0.26
Fig. 6d	-	0.91	-	0.09
Fig. 8a(i)	0.48	0.09	0.43	
Fig. 8a(ii)	0.37	0.18	0.45	
Fig. 8a(iii)	0.21	0.39	0.40	
Fig. 8a(iv)	0.11	0.56	0.33	
Fig. 8a(v)	0.09	0.91	-	
Fig. 8b(i)	0.85	0.15	-	-
Fig. 8b(ii)	0.82	0.18	-	-
Fig. 8b(iii)	0.35	0.65	-	-
Fig. 8b(iv)	0.18	0.82	-	-
Fig. 8b(v)	0.08	0.92	-	-

Table S1: Normalised UPLC peak areas from the CuAAC trapping experiments shown in Figure 6 and Figure 8. For the UPLC traces shown in Figure 8(a), it was not possible to reliably deconvolute the macrocyclic duplex and linear duplex peaks, so peak areas are reported as a combined value.

Azide Reactivity Test

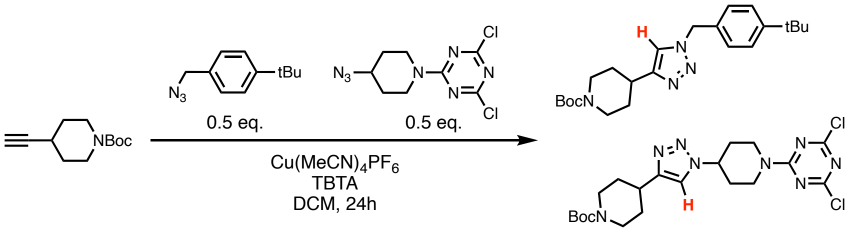

Figure S25: Model reaction to test whether there is any difference in the reactivity under CuAAC conditions of 4-*t*-butylbenzyl azide and **5**.

It was necessary to check whether there was difference in the rate of the CuAAC reaction between the terminal azide of an oligomer with 4-*t*-butylbenzyl azide vs the terminal azide of another oligomer, as this would affect the distribution of species observed in the CuAAC trapping experiments. To test this, a model reaction was carried out.

To a solution of 1-Boc-4-ethynylpiperidine (10 mg, 0.048 mmol), 4-*t*-butylbenzyl azide (4.5 mg, 0.024 mmol) and **5** (6.6 mg, 0.024 mmol) in dry DCM (2 mL) was added a solution of $Cu(CH_3CN)_4PF_6$ (7.5 mg, 0.02 mmol) and TBTA (11 mg, 0.02 mmol) in dry DCM (3 mL) under N_2 atmosphere. The reaction was stirred overnight at r.t. before the solvent was removed *in vacuo*. The mixture was extracted with EtOAc (3x) and washed with 0.01 M EDTA solution (2x) and brine. The organic phase was dried over $MgSO_4$ and then the solvent was removed *in vacuo* to yield the product mixture.

Figure S26 shows that the two triazole proton signals (7.99 and 7.95 ppm) in the ^1H NMR spectrum (DMSO-d$_6$) of the product mixture both integrate to one – i.e. there is no difference in rate of reaction between the two azide species.

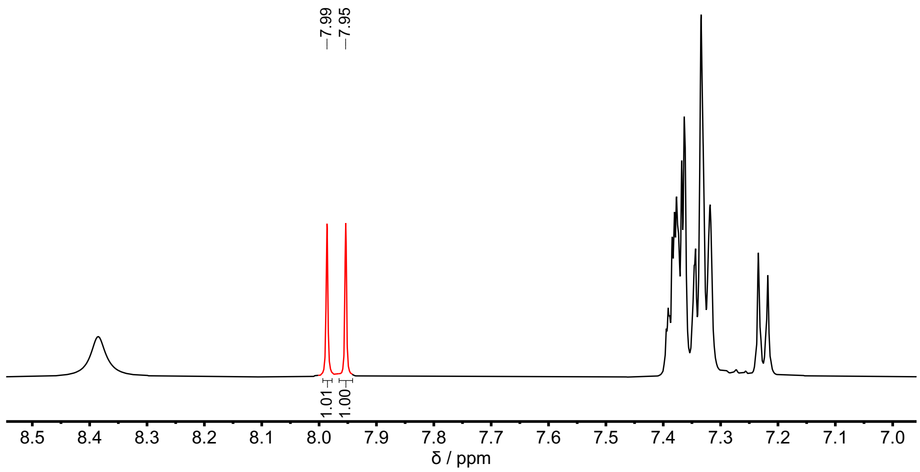

Figure S26: Downfield region of the ^1H NMR (400 MHz, DMSO-d$_6$, 298 K) spectrum of the product distribution after the reaction shown in Figure S26.

HPLC Separation and Characterisation of zDADAy CuAAC Products

To further confirm the identity of the products displayed in Figure 6, the **zDADAy** (1 mM) CuAAC trapping experiment was repeated on a 10 mg scale. The major products were separated and isolated by HPLC, using the same HPLC conditions as described in Section 4.

A gradient of 57.5% B over 40 mins, then 57.5 to 65% B over 20 mins was used to separate the three major products.

HRMS (ES+): linear single strand: calculated for $C_{95}H_{148}N_{30}O_4P_2$ 1837.1876 $[M+H]^+$, found 1837.1900 $[M+H]^+$; **macrocyclic duplex:** calculated for $C_{168}H_{266}N_{54}O_8P_4$ 1648.0610 $[M+2H]^{2+}$, found 1648.0628 $[M+2H]^{2+}$; **linear duplex:** calculated for $C_{179}H_{281}N_{57}O_8P_4$ 1764.6062 $[M+2Na]^{2+}$, found 1764.5980 $[M+2Na]^{2+}$.

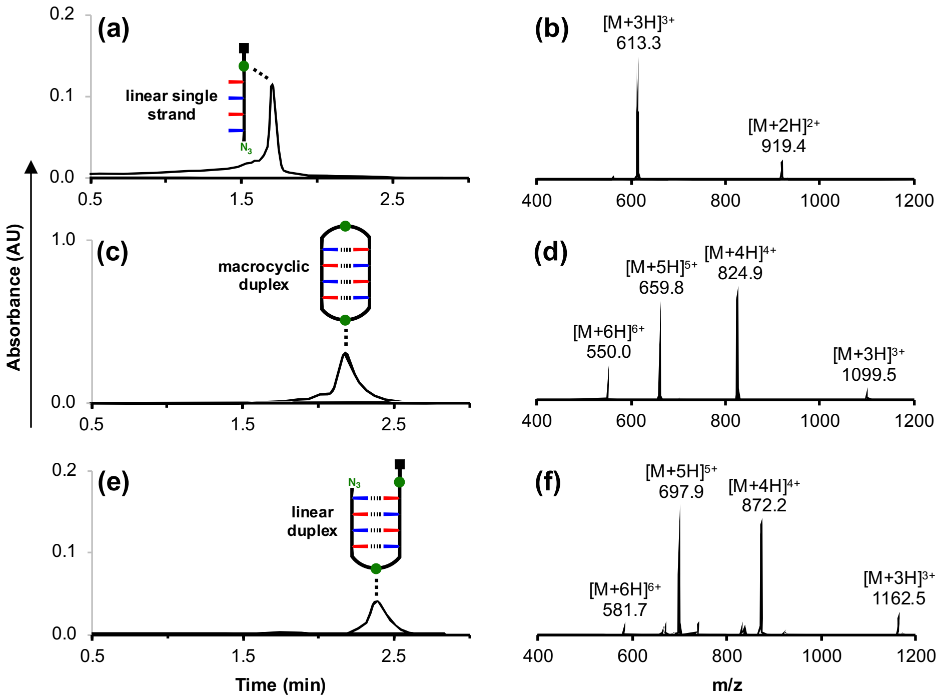

Figure S27: Isolated products from the CuAAC reaction of **zDADAy** (1 mM) and 4-*t*-butylbenzyl azide (1 mM). **(a)** UPLC trace of linear single strand. **(b)** ESI-MS of linear single strand. Calculated mass (ESI^+): 919.2 $[M+2H]^{2+}$, 613.4 $[M+3H]^{3+}$. **(c)** UPLC trace of macrocyclic duplex. **(d)** ESI-MS of macrocyclic duplex. Calculated mass (ESI^+): 1099.4 $[M+3H]^{3+}$, 824.8 $[M+4H]^{4+}$, 659.8 $[M+5H]^{5+}$, 550.0 $[M+6H]^{6+}$. **(e)** UPLC trace of linear duplex. **(f)** ESI-MS of linear duplex. Calculated mass (ESI^+): 1162.4 $[M+3H]^{3+}$, 872.1 $[M+4H]^{4+}$, 697.9 $[M+5H]^{5+}$, 581.7 $[M+6H]^{6+}$. *UPLC Conditions:* C4 column at 40 °C (254 nm) using a 65-100% gradient of MeCN/formic acid (0.1%) in water/formic acid (0.1%) over 2 minutes, then 100% MeCN/formic acid (0.1%) over 1 minute.
